# ANZAED 2023 Gold Coast Conference: Oral & Poster Abstracts

**DOI:** 10.1186/s40337-024-01046-4

**Published:** 2024-07-16

## O1 Examining the effectiveness of the Eating Disorder Credential training and skill development pathway: Testing a framework for large-scale skill development in eating disorders

### *Emma Spiel^1^, Sarah Trobe^1^, Beth Shelton^1^, Gabriella Heruc^2^, Chanel Agosta^2^, Noni O'Dea^2^, Kim Hurst^2,4^, Siân McLean^2,3^

#### ^1^NEDC, Crows Nest, NSW, Australia; ^2^ANZAED, St Leonards, NSW, Australia; ^3^La Trobe University, Bundoora, VIC, Australia; ^4^Robina Private, Robina, QLD, Australia

##### Correspondence: emma.spiel@nedc.com.au

*Journal of Eating Disorders* 2024, 12(suppl 2): O1

Despite ongoing efforts to improve service reach and capacity across Australia, people experiencing eating disorders, their loved ones, and communities report ongoing difficulties in accessing timely, effective care. Problems with accessing care are due in part to a shortage of a skilled workforce large enough in size to meet Lived Experience needs. A framework for upskilling large numbers of clinicians in an evidence-based treatment approach that could help address workforce shortages was developed as part of the ANZAED Eating Disorder Credential system. This research aims to determine the effectiveness of the program in relation to participants’ perceived skill, confidence, and willingness to provide treatment for people experiencing eating disorders.

Methods: Participants include up to n = 200 mental health professionals and dietitians who completed a funded professional development package (PDP) of training and supervision as part of the ANZAED Eating Disorder Credential system. Survey data collected prior to participants receiving their PDP and following completion of their PDP will assess changes in perceived competency and attitudes towards provision of eating disorder treatment.

Results: Demographic data will be presented regarding key aspects of current clinician practice including work setting, location, diagnostic presentations, and experience providing treatment for people experiencing eating disorders. Finally, data will be presented regarding changes in clinician perceived skill, confidence, and willingness to provide treatment for people experiencing an eating disorder having undertaken the program.

Conclusions: Implications for the future workforce and challenges and opportunities for use of large-scale eating disorder workforce development initiatives will be discussed.

## O2 Body Image Beliefs and the Metacognitive Model

### *Esben Strodl^1^

#### Queensland University of Technology, Brisbane, QLD, Australia

##### Correspondence: e.strodl@qut.edu.au

*Journal of Eating Disorders* 2024, 12(suppl 2): O2

While body image beliefs are widely understood to be important in the genesis and perpetuation of eating disorders, they are also notoriously difficult to change. In addition, the literature points towards the presence of individual differences moderating the strength of the associations between antecedents and consequences of body image concerns. New theoretical frameworks for understanding the nature of body image beliefs are therefore needed to guide the development of novel interventions. This presentation will argue that the metacognitive model provides a useful theoretical framework to understand the individual factors involved in driving the potency of body image beliefs and their resistance to change. This will be illustrated by reinterpreting examples of body image research through a metacognitive model lens. In addition, the presentation will draw upon recent data from an online survey of 740 Australian young adults to illustrate the application of the metacognitive model to the link between body dissatisfaction, drive for thinness and restrained/bulimic eating behaviours. Finally, the presentation will provide suggestions for the application of the metacognitive model to interventions for body image concerns and eating disorders.

## O3 Eating disorder presentations and practice considerations for fertility care

### *Hannah Lack^1^, Hilary Smith^2^, Sarah Trobe^1^, Christie Bennett^2^

#### ^1^National Eating Disorder Collaboration, Crows Nest, NSW, Australia; ^2^Monash University, Clayton, VIC, Australia

##### Correspondence: info@nedc.com.au

*Journal of Eating Disorders* 2024, 12(suppl 2): O3

Background and aim: There is a higher prevalence of eating disorders in people seeking fertility treatment. As such, the intersection of eating disorders and fertility care and the role of fertility care professionals in responding to eating disorders warrants exploration. The aim of the current project was to conduct a rapid review of the prevalence, screening, and presentations of disordered eating and eating disorders in fertility care treatment settings, and to summarise key challenges in the attitudes and knowledge of fertility care professionals. A strategy to better equip this workforce to prevent, identify, and respond to eating disorders within their scope of practice is proposed. Methods: A rapid literature review was conducted to explore the intersection and presentation of eating disorders, body image issues and disordered eating in the fertility are setting and recommendations from an expert advisory group (EAG) were summarized to identify common knowledge and attitudinal gaps in the fertility care workforce.

Results: The rapid review and feedback from the EAG highlighted several key knowledge themes: increased prevalence of EDs in people accessing fertility care, the negative impact of EDs on reproductive health and fertility outcomes, and the risk factors associated with infertility and fertility treatment on experiences of body image issues, disordered eating and eating disorders.

Conclusion: The research highlighted the clinical issue of eating disorders in fertility care settings. Findings will be used to inform the development of tools to address common knowledge and skill gaps in the fertility care workforce to address this issue.

## O4 The NDIS—improving engagement for people with eating disorders

### *Hilary Smith^1^

#### ^1^National Eating Disorders Collaboration, Crows Nest, NSW, Australia

##### Correspondence: hilary.smith@nedc.com.au

*Journal of Eating Disorders* 2024, 12(suppl 2): O4

Since its inception in 2013, the National Disability Insurance Scheme (NDIS) has grown to support over 484,000 Australians. Intended for people with permanent and significant disability, the position of people with mental health conditions within the NDIS policy framework has been the subject of debate for much of the period of the Scheme’s design and operation. This has led to a lack of clarity as to when a person with a mental health condition, or psychosocial disability, may be considered eligible for the NDIS. This includes people with eating disorders. In 2022–23, the Australian Government Department of Health and Aged Care commissioned the National Eating Disorders Collaboration (NEDC) to compile a summary of key issues for the engagement of people with eating disorders with the NDIS, and to consider opportunities to improve this engagement. NEDC undertook a policy review as well as engagement with key stakeholders within the eating disorder and disability sectors and government in order to compile the summary. The summary includes examination of issues for people whose primary diagnosis is a severe and enduring eating disorder, as well as for NDIS participants whose eligibility is associated with another disability and who also experience an eating disorder. Themes relating to NDIS access and engagement, such as lack of policy clarity and workforce issues, are illustrated by use of case studies demonstrating both positive and negative engagement experiences. Key learnings and proposed mechanisms for improvement are presented.

## O5 Correlations between Videoconferencing Use, Objectification Processes, Depression and Eating Disorder Risk

### *Jade Portingale^1^, An Dang^1^, Isabel Krug^1^

#### ^1^University of Melbourne, Melbourne, VIC, Australia

##### Correspondence: jportingale@student.unimelb.edu.au

*Journal of Eating Disorders* 2024, 12(suppl 2): O5

Background: Videoconferencing for work/study has been linked to body image concerns, eating pathology, and depression. However, prior research has neglected the role of trait variables, such as those affecting an individual’s appraisal of their appearance during social interactions, in these relationships. Objective: The current study examined for the first time whether videoconferencing usage frequency moderated the association between appearance-based rejection sensitivity (appearance-RS), self-objectification, and body-ideal internalisation with disordered eating (DE; total; oral control; bulimia/food preoccupation; dieting), body dissatisfaction, and depressive symptoms Method: A total of 1,615 Australia-based participants (76.8% women) took part in an online survey assessing demographic and trait-level psychological variables. Across the sample, 1,201 (74.4%) were classified as high-frequency users (based on current usage of multiple times a week or more) and 414 (25.6%) were low-frequency users (based on current usage less than multiple times a week or not at all). Results: Across the entire sample, high (relative to low) frequency videoconferencing usage predicted greater DE symptoms and greater body dissatisfaction but did not predict depressive symptoms. Videoconferencing usage frequency moderated the effect of self-objectification on DE (oral control) symptoms and the effect of body-ideal internalisation on body dissatisfaction: however, failed to moderate the remaining effects. Discussion: Our findings highlight that videoconferencing platforms may reflect an environment conducive to increased ED-related risk, particularly for high-frequency users and those with a tendency towards self-objectification and body-ideal internalisation. Future replication and extension are encouraged.

## O6 Embodiment Illusions May Elucidate Transdiagnostic Factors Across Disordered Eating, Psychosis, Depression, and Anxiety: A Systematic Scoping Review

### *Jade Portingale^1^, David Butler^2^, Cali Bartholomeusz^1^, Tamsyn Van Rheenen^1^, Litza Kiropoulos^1^, Isabel Krug^1^

#### ^1^University of Melbourne, Melbourne, VIC, Australia

##### Correspondence: jportingale@student.unimelb.edu.au

*Journal of Eating Disorders* 2024, 12(suppl 2): O6

Background: Researchers have increasingly used embodiment illusions to understand and improve various mental disorders, separately. However, the capacity for these illusions to shed light on transdiagnostic constructs or underlying mechanisms shared across mental disorders remains largely unclear. Objective: This systematic scoping review is the first to synthesise and appraise the literature examining embodiment illusions as a function of (Aim 1), and method to improve (Aim 2), symptoms of disordered eating, psychosis, depression, and anxiety (including symptom spectrums and categorical diagnoses). Method: PsycINFO, MEDLINE, EMBASE, Web of Science, and CINAHL were searched for published articles until December 2022. Results: Forty-nine studies met the inclusion criteria. Across the 33 studies relevant to Aim 1, greater susceptibility to embodiment illusions was predominately observed among those with high (relative to low/no) symptoms of disordered eating (82%), psychosis (95%), and depression (33%); no effects were found for anxiety. Across the 22 studies relevant to Aim 2, improvement in symptoms post-embodiment were observed in the majority that assessed disordered eating (83%), depression (100%), and anxiety (75%); no studies assessed psychosis. Discussion: Current findings (i) highlight distorted bodily-self-perception (likely underpinned by impaired multisensory integration) as a potentially common factor underlying psychosis, disordered eating, and preliminarily, depression; and (ii) support the use of embodiment illusions in treating specific symptoms of disordered eating, depression, and anxiety. Future research into the development of transdiagnostic programs aimed at improving bodily distortions via multisensory integration deficits is critical. Future researchers should also explore whether embodiment illusions can improve psychosis.

## O7 Australian Elite Sport Coaches’ Mental Health Literacy of Eating Disorders in Athletes: A Qualitative Study

### Jardine Mitchell^1^, Litza Kiropoulos^1^, Molly Tilbrook^1^, *Isabel Krug^1^

#### ^1^The University of Melbourne, Melboure, VIC, Australia

##### Correspondence: isabel.krug@unimelb.edu.au

*Journal of Eating Disorders* 2024, 12(suppl 2): O7

Background: Athletes have an increased risk of developing an eating disorder compared to the general population, particularly in sports which emphasize body ‘ideals’. Sport coaches are in a unique position to identify eating disorder symptoms and promote timely support in athletes. However, sport coaches’ mental health literacy of eating disorders remains understudied. This study aimed to qualitatively investigate Australian elite sport coaches’ mental health literacy of DSM-5 eating disorders, including emerging Orthorexia and Bigorexia conditions, in athletes.

Methods: Eighteen Australian elite sport coaches from at-risk sports participated in a semi-structured interview to understand their knowledge of eating disorders, beliefs about managing eating disorders in elite sport, and suggestions for future eating disorder training resources. Interview data was analysed using reflexive thematic analysis and four main themes emerged.

Results: Coaches shared: (1) limited knowledge of the psychological signs of eating disorders, eating disorders without observable weight loss, and Orthorexia; (2) facilitators to managing concerns including the athlete-coach relationship, professional supports, using functional rather than appearance-related language, and lived experience; (3) barriers including hesitancy to communicate about body image and food, responding to denial, and accessing support services; (4) suggestions for in-person, experiential eating disorder training embedded into coaching accreditation.

Implications and Conclusion: This study has practical implications for developing eating disorder-specific education and training resources for coaches. Our findings highlight the need for multidisciplinary input when developing and delivering training, as well as implementing communication-based training opportunities to increase coach confidence to identify and manage eating disorders in their athletes.

## O8 Coaches knowledge, attitudes, beliefs and/or behaviours towards eating disorders and low energy availability

### *Dr Jennifer Ellen Hamer ^1^, Prof Ben Desbrow ^1^, Dr Chris Irwin^1^

#### ^1^Griffith University, Southport, QLD, Australia

##### Correspondence: jennifer.hamer@griffithuni.edu.au

*Journal of Eating Disorders* 2024, 12(suppl 2): O8

**Link**—A review exploring coach knowledge, attitudes/beliefs and behaviours towards low energy availability in athletes—Jennifer Hamer, Ben Desbrow, Chris Irwin, 2023 (sagepub.com). https://journals.sagepub.com/doi/abs/10.1177/17479541221140188.

## O9 The Effectiveness of Peer Mentoring for Eating Disorder Recovery: A 10-Year Review of Eating Disorders Queensland’s Peer Mentoring Program for Mentees and Mentors

### *Jessica Tone^1^, Kayla Lazzam^1^, Belinda Chelius^1,2^

#### ^1^Eating Disorders Queensland, South Brisbane, QLD, Australia; ^2^School of Public Health and Social Work, Queensland University of Technology, Kelvin Grove QLD, Australia

##### Correspondence: jesst@edq.org.au

*Journal of Eating Disorders* 2024, 12(suppl 2): O9

Eating Disorders Queensland (EDQ) piloted a peer mentor program (PMP) in 2012 and has since implemented the program biannually over the past 10 years. PMP supports people who are recovering from an eating disorder (ED) by partnering mentees who are motivated towards recovery, with volunteer mentors who are in two-year stable recovery from an ED. The mentoring role has been integrated into an organisational lived experience practice framework that supports growth opportunities for paid lived experience positions. Current research into PMPs for EDs indicates preliminary benefits for mentee recovery, however, there remain gaps in the evaluation of mentor outcomes and limited discussion around mentor training and supervision. The aim of this evaluation is to review and discuss findings from EDQ’s PMP over the past decade to provide an update on the effectiveness of the program for mentees, monitoring of mentor outcomes, training and supervision, the volunteer role, and overall learnings and adaptions of the program based on consumer feedback. Evaluation of programs run until 2021 indicate improvements in the RAS-DS, EDE-Q, and DASS-21 for mentees (n = 74) and stable outcomes for mentors (n = 41). We will present updated findings for mentees and mentors collected through routine quantitative and qualitative analysis of mentee/mentor outcomes and feedback provided by participants. Our aim is to contribute to the growing body of literature examining PMP benefits for mentees and begin to address current gaps around experiences for mentors.

## O10 Reducing inpatient care in Paediatric Eating Disorders: A Retrospective Review of Outcomes: Paediatric Eating Disorders Hospital in the Home

### *Jo Centra^1^

#### ^1^Barwon Health, Barwon, VIC, Australia

##### Correspondence: johanna.centra@barwonhealth.org.au

*Journal of Eating Disorders* 2024, 12(suppl 2): O10

The innovative Paediatric Eating Disorders Service Hospital in the Home (HITH) program was designed to address the increased prevalence of paediatric eating disorders during the Covid-19 pandemic; the difficulty accessing prompt and effective treatment and the increased need for long hospital admissions due to high patient acuity. The HITH program aims to maintain the same weight gain success obtained by an inpatient admission, but with reduced burden of inpatient care and myriad associated benefits. The study compares the HITH model with routine inpatient hospital admission for unwell paediatric patients with an eating disorder. This study is a retrospective data comparison of two patient groups: ‘case’- during HITH study period, and ‘control’- prior to HITH study implementation, over two comparable 6 month periods.Results confirm comparable clinical effectiveness of the HITH program with average weight gain over the initial two weeks of HITH admission: 2.4kg, compared with the first two weeks of inpatient admission: 2.6kg. Secondary outcomes reflect reduced need for inpatient admission with the implementation of HITH, inpatient bed days of 141 during study period (HITH in place) and 264 during control period. The HITH program has the potential to radically change the way we care for paediatric patients with an eating disorder, leading to a substantial reduction in the time spent as an inpatient; reducing the need for protracted, expensive inpatient hospital admissions and facilitating safe and effective patient and family-centred care.

## O11 The supervision experience: How clinicians view the ANZAED Eating Disorder Credential’s ongoing professional development

### *Katarina Prnjak^1,2^, Gabriella Heruc^1,3,4^, Janet Conti^1^, Deborah Mitchison^1,2,5^, Siân McLean^6^, Noni O'Dea^1^, Rebecca Barns^1^, Chanel Agosta^1^, Phillipa Hay^1,7^

#### ^1^Western Sydney University, Penrith, NSW, Australia; ^2^University of Technology Sydney, Sydney, NSW, Australia; ^3^ANZAED, Ste Leonards, NSW, Australia; ^4^Appetite for Change, Crows Nest, NSW, Australia; ^5^Basten & Associates, Sydney, NSW, Australia; ^6^La Trobe University, Melbourne, VIC, Australia; ^7^Camden and Campbelltown Hospitals, SWSLHD

##### Correspondence: katarinaprnjak@gmail.com

*Journal of Eating Disorders* 2024, 12(suppl 2): O11

The ANZAED Eating Disorder Treatment Principles and General Clinical Practice and Training Standards and the recently launched ANZAED Eating Disorder Credential both endorse the importance of eating disorder (ED) treatment providers undertaking clinical supervision as part of their ongoing professional development. However, there is currently no nationwide framework of ED-specific supervisory standards. This mixed-methods study aimed to explore experiences of Credentialed Eating Disorder Clinicians receiving and providing ED-specific supervision. N = 220 clinicians (92% women; Mage[SD] = 38.37[10.14], with M[SD] = 11.24[8.14] years of experience) across Australia, comprising 41% psychologists, 40% dietitians, 9% social workers and 10% other professionals, completed an online survey in February and March 2023, with n = 22 participating in one-on-one interviews. Most participants (61%) received supervision only, 4% provided supervision only, and 35% undertook both. The majority (88% of supervisees and 85% of supervisors) reported their experience of supervision as “good” or “excellent.” Supervisees most strongly endorsed that supervision provided a safe and welcoming space to reflect on their practice, and 67% agreed that the ongoing supervisory requirements for maintaining the Credential were reasonable. Of concern were the most commonly reported barriers towards receiving supervision: lack of time (65%) and cost (60%). Overall, 71% of supervisors felt well-equipped to provide ED-specific supervision, whereas 81% and 80% reported seeing improvements in confidence and competence of their supervisees, respectively. These preliminary findings show that supervision was perceived as beneficial for improving clinician confidence and competence to deliver treatment, and indicate potential avenues for improving supervisees’ experience.

## O12 Perceived impact of the ANZAED Eating Disorder Credential on treatment delivery: Integrating perspectives of clinicians, individuals with eating disorder lived experience, and carers

### *Katarina Prnjak^1,2^, Gabriella Heruc^1,3,4^, Janet Conti^1^, Deborah Mitchison^1,2,5^, Siân McLean^6^, Noni O'Dea^1^, Rebecca Barns^1^, Chanel Agosta^1^, Phillipa Hay^1,7^

#### 1. Western Sydney University, Penrith, NSW, Australia2. University of Technology Sydney, Sydney, NSW, Australia3. ANZAED, St Leonards, NSW, Australia4. Appetite for Change, Crows Nest, NSW, Australia5. Basten & Associates, Sydney, NSW, Australia6. La Trobe University, Bundoora, NSW, Australia7. Camden and Campbelltown Hospitals, NSW, Australia

##### Correspondence: katarinaprnjak@gmail.com

*Journal of Eating Disorders* 2024, 12(suppl 2): O12

The ANZAED Eating Disorder Credential formally recognises clinicians with qualifications, knowledge, training, and ongoing professional development (PD) to meet minimum standards for the delivery of safe and effective eating disorder (ED) treatment. This study aimed to investigate how the Credential is perceived and valued by Credentialed Eating Disorder Clinicians. N = 220 clinicians (92% women; Mage[SD] = 38.37[10.14], with M[SD] = 11.24[8.14] years of experience) across Australia, comprising 41% psychologists, 40% dietitians, 9% social workers and 10% other professionals, completed an online survey in February and March 2023. Around 25% of participants indicated that being credentialed had increased the number of ED clients they treat. In general, participants rated their experience engaging with the Credential as positive. Considering the importance of ongoing PD, 80% reported that it enhanced their understanding of ED assessment and management, increased their confidence, and helped them acquire new skills. Furthermore, 70% reported that it increased their willingness to treat EDs, and 68% reported that PD requirements for maintaining the Credential were reasonable. Notably, the most highly valued aspect of the Credential was that it provides safety, confidence and trust for treatment seekers (87% responded “rather much” or “very much”). Of the 66 participants who have searched for a clinician using the ‘Find a treatment provider’ directory, 57 found it useful for identifying a clinician who is a right fit for their client. Overall, these preliminary findings indicate that the Credential was perceived by clinicians as beneficial across various domains of their work with ED clients.

## O13 An evaluation of the Door 2 program as an approach to understanding and treating Eating Disorders in Neurodivergent populations

### *Kelsey Sherwood^1^, Dr Nerelie Freeman^1^

#### ^1^Monash University, Melbourne, VIC, Australia

##### Correspondence: kelsey@door2.com.au

*Journal of Eating Disorders* 2024, 12(suppl 2): O13

Current research estimates up to 30% of people with an eating disorder (ED) also have autism or autistic traits. The need for more intensive, nuanced and tailored supports will help address the increased likelihood of poorer treatment outcomes experienced by this cohort. However, individualized treatment that helps understand EDs in the context of neurodiversity is in its developmental infancy. The Bronte Centre online Door 2 program is one of few available supports addressing the specific needs of this cohort and their families. Semi-structured interviews (mean duration = 98 min) were conducted using Zoom videoconferencing with nine mothers of daughters aged 13 – 19 years old (mean = 16.44 years) with an ED and autistic traits asking about their experience participating in this program. Interpretative phenomenological analysis was used along with member checks to derive themes from the participants’ narratives. Two superordinate themes emerged from the interviews. “Taking a step back” consisted of narratives around the parent–child relationship and “We needed to do something different” consisted of narratives around past and present support parents have received. Recognition of the co-occurrence (ED and autism) and access to lived experience mentors were considered important components in treatment of the ED. Limitations included the positionality of the student researcher, the brief recruitment period, and the composition of the sample being all Caucasian mothers. While further research is needed to evaluate the effectiveness of the Door 2 program, findings suggest it is a promising support option for families of young people with EDs and autistic symptoms.

## O14 Examining efficacious outcomes of an Eating Disorder Service

### *Kim Hurst^1,2^, Aimee Maxwell^1^, Melissa Marks^1^, Melissa Cheras^1^, Amanda Durham^1^, Rachel Ivey^1^, Monique Jephcote^1^, Caitlin Gleeson^1^, Jonothan Hespe^1^, Sonja Kubik^1^, Vinay Garbharran^1^, Emma Phelan^1^, Kana Ichikawa^1^, Laura Vornanen^1^

#### ^1^Robina Private Hospital Eating Disorders Service, Robina, QLD, Australia; ^2^School of Applied Psychology, Griffith University, Gold Coast, QLD, Australia

##### Correspondence: kim.hurst@aurorahealth.com.au

*Journal of Eating Disorders* 2024, 12(suppl 2): O14

Intervention for eating disorders should match the complexity and acuity of the illness. The goal of this study contributes to evidence base practices regarding the efficacy of inpatient and outpatient programs for treating eating disorders. 325 patients were admitted to Robina Private Hospital’s Eating Disorder Service from October 2019—November 2022, 203 were inpatients; 122 outpatients. Anorexia Nervosa was the primary diagnosis for inpatient (67.48%) and outpatient (72.13%). The average length of stay for inpatients was 31.90 days (SD = 23.09) compared to outpatients 20.21 days, SD = 11.44. 44 inpatients (21.68%) transitioned to outpatient programs, whilst 10 outpatients (8.2%) required admission to inpatient programs. For inpatients, 163 (80.30%) were first admissions compared to 90 (75%) for outpatients, 25 inpatients had a second admission (12.31%) compared to 22 outpatients (18.33%). 15 inpatients recorded 3 + admissions (7.39%), compared to 8 outpatients (6.67%), of these 13 inpatients (86.6%) had complex mental health conditions, including personality disorders and PTSD, compared to 74.97% in outpatient. BMI significantly differed between pre (M = 18.92, SD = 3.64) and post (M = 19.99, SD = 3.22, t(190) = -13.4, two-tailed p < 0.001) for inpatients. T-tests showed significant decrease in pre (M = 4.20, SD = 1.20) and post (M = 3.26, SD = 1.21) EDE-Q scores, t(44) = 4.50, two-tailed p < 0.001. Similar results were recorded for the depression subscales (Mpre = 12.09, SDpre = 4.81; Mpost = 10.65, SDpost = 5.34,, t(37) = 2.96, two-tailed p < 0.01) of the DASS-21. Additional outcomes measures provide preliminary support for the efficacy of the Robina Private Eating Disorder Service in improving patient outcomes.

## O15 How adolescence perceive parental comments on weight shape and eating and associations with eating disorders over time, a mixed-method study

### *Lucy M Dahill^1^, Natalie M.V. Morrison^1^, Haider Mannan^1^, Stephen Touyz^2,3^, Deborah Mitchison^1,4^, Kay Bussey^5^, Phillipa Hay^1,6^

#### ^1^Translational Health Research Institute, School of Medicine, Western Sydney University, Sydney, NSW, Australia; ^2^University of Sydney Inside Out, Sydney, NSW, Australia; ^3^Sydney Local Health District, Sydney, NSW, Australia; ^4^University of Technology, Ultimo, NSW, Australia; ^5^Macquarie University, Centre for Emotional Health, Ryde, NSW, Australia; ^6^South West Sydney Local Health District, Camden and Campbelltown Hospitals, Campbelltown, NSW, Australia

##### Correspondence: L.Dahill@westernsydney.edu.au

*Journal of Eating Disorders* 2024, 12(suppl 2): O15

Disordered eating amongst adolescents is of increasing concern given associated physical and mental health sequalae. Parents are a significant presence during this period and make more positive than negative comments on weight shape and eating, so it is critical to understand how they influence their adolescent’s eating cognitions and behaviours and consider how they hear the words their parents are saying. This mixed method review considers associations of eating and weight shape comments with parental positive and negative comments over time (n = 2056). Results indicated that positive maternal comments on eating were associated with increased EDC’s and better quality of life at one year. Paternal positive weight shape comments were associated with a decrease in psychological distress, but positive eating comments saw a decrease in quality of life. Qualitative analysis from the same cohort (N = 10, 60% female) add colour and meaning to these statistics “a slight negative comment would feel more intense than the positive comment that weighs three times as much.” Through the lens of Bronfenbrenner and Morris’ 2007 Bioecology Theory and using Interpretative Phenomenological Analysis we considered their lived experiences as they engaged with their parents in a range of weight, shape, and eating communications. We found the adolescents whose parents had reported lower tolerance for humour expressed more positive body-esteem; and a bi-directional fear of ‘saying the wrong thing’ led to potential dangers through family loyalty and in-group permissions. The mixed-method approach offers a rich illustration of adolescents’ experiences and meaning-making of parental weight shape and eating commentary.

## O16 Co-designing a brief intervention and evaluating its feasibility for use in Community Child Health Services

### *Lyza Norton^1^, Joy Parkinson^1, 2^, Neil Harris^1^, Margaret MacGuinness^3^, Laura Hart^4^

#### ^1^Griffith University, QLD, Australia; ^2^CSIRO, VIC, Australia’; ^3^Gold Coast Hospital and Health Service, QLD, Australia; ^4^The University of Melbourne, VIC, Australia

##### Correspondence: lyza.norton@griffithuni.edu.au

*Journal of Eating Disorders* 2024, 12(suppl 2): O16

How parents talk about food with their infants is important for developing positive child eating behaviours. In Australia, Child Health Nurses provide community-based education to new parents. However, limited information is presented to parents on “how” they can communicate with their infants to promote healthful eating behaviours. Research highlights that the conversations parents have during mealtimes influences children’s eating habits into the future. This presentation will describe how co-design workshops with Child Health Nurses were used to translate research on parent food communication into an education session for parents. It will also outline how the resulting intervention was evaluated. First, employing the Knowledge to Action Framework, Child Health Nurses (n = 15) attended co-design workshops to develop: “Mealtime Chatter Matters” (MCM). The MCM session focused on developing knowledge of evidenced-based, practical strategies for parents to talk with their children during mealtimes. To test the acceptability of the program and to gather initial information about the impact of the intervention on parents’ feeding behaviour, an uncontrolled repeated measures pilot trial was conducted (n = 46 parents, n = 6 Nurses). Acceptability was assessed via questionnaires and semi-structured interviews with a subset of parents (n = 9). Impact was measured via pre and post self-reported questionnaires. Findings indicated high acceptability of the MCM program by nurses and parents. Findings for impact, however, were unclear and require further investigation. Our study led to important lessons for the future measurement of parental food communication and the conduct of feasibility studies within the eating disorder prevention space.

## O17 Implementation of FBT-informed Interdisciplinary Model of Care in an inpatient medical setting to improve outcomes for young people admitted with restrictive eating

### *Martha Churchett^1^, Michelle Boyd^1^, Elise Burn^1^

#### ^1^Queensland Children's Hospital, Brisbane, QLD, Australia

##### Correspondence: martha.churchett@health.qld.gov.au

*Journal of Eating Disorders* 2024, 12(suppl 2): O17

Queensland Children’s Hospital (QCH) has recorded a significant increase in presentations of young people with restrictive eating. Subsequently, QCH implemented an interdisciplinary team consisting of a paediatrician, clinical nurse consultant, and dietitian with a short-term goal of reducing hospital readmission underpinned by increased wait time for public and private outpatient services. This team, known as EDAT (Eating Disorder Acute Team) has implemented an FBT informed model of care in the inpatient medical setting that has demonstrably improved outcomes for young people by reducing hospital readmission rates whilst preparing families for outpatient treatment. EDAT, through an interdisciplinary approach, supports families through the medical admission by offering high quality and consistent management through psychoeducation and introduction of FBT principals early in the patient journey. Application of FBT principles within inpatient medical treatment assists in streamlining transition to community outpatient evidence-based treatment. This is completed by intensive education completed by the care team in the form of weekly ward rounds, education sessions using FBT principles of meal support and discharge discussions focusing on parental empowerment for readiness to commence FBT in an outpatient space. Additionally supporting the broader QCH Team to provide the FBT informed model of care to nursing, medical and allied health staff. EDAT has successfully reduced re-admission rates and most importantly increased parental confidence through a change in MOC, aligning goals of treatment amongst inpatient and outpatient services. In addition to providing a cohesive transfer of care into the community.

## O18 Taking a Closer Look at OSFED: A Systematic Review and *Meta*-Analysis of Psychopathology of Diagnostic Subgroups

### Max Dang^1^, Elizabeth Hughes^1^, An Dang^1^, Heung Ying Lai^1^, Jessica Lee^1^, Shanshan Liu^1^, Jade Portingale^1^, *Isabel Krug^1^

#### ^1^The University of Melbourne, VIC, Australia

##### Correspondence: isabel.krug@unimelb.edu.au

*Journal of Eating Disorders* 2024, 12(suppl 2): O18

Other Specified Feeding and Eating Disorder (OSFED) is a highly prevalent diagnosis, yet little is known about how each diagnostic subgroup within OSFED compares to other full-threshold eating disorders (EDs). Understanding this would help improve diagnostic classification and inform guidelines for clinicians working with OSFED patients. The current review compared all OSFED subgroups (Atypical Anorexia Nervosa [AAN], Subthreshold Bulimia Nervosa [Subthreshold-BN], Subthreshold Binge Eating Disorder [Subthreshold-BED], Purging Disorder [PD], and Night Eating Syndrome [NES]) to full-threshold EDs (Anorexia Nervosa [AN], Bulimia Nervosa [BN], and Binge Eating Disorder [BED]) and healthy individuals on dimensions of eating and general psychopathology. Following a comprehensive literature search across four electronic databases from January 2013 to March 2023, 29 studies were included in the systematic review and 31 studies qualified for the meta-analysis of group differences on global measures of eating psychopathology. Our systematic review found that, generally, individuals across all OSFED subgroups exhibited a) higher eating and general psychopathology than healthy individuals, and b) similar eating and general psychopathology to full-threshold EDs. Our meta-analysis results supported these findings and provided relevant data for AAN, PD, and NES. There was a notable lack of research for Subthreshold-BN and Subthreshold-BED. Overall, this review supports the conceptualisation of OSFED subgroups as a clinically significant EDs with similar severity to full-threshold EDs, in particular for AAN, PD and NES. However, limited data in the current literature means that more research examining aspects of psychopathology of OSFED is required to inform changes to diagnostic classifications and guide clinical work.

## O19 Building support for clinicians across a large, geographically diverse Local Health District: Matching clinician-reported need with tailored training and support

### *Melissa Hart^1,2,3^, Katie Rolfe^3^, Jemma Olson^3^, Leonnie Moore^3^, Anjanette Casey^3^, Annette Murphy^3^, Claire Toohey^3^, Julie Mavay^3^, Matthew Elton^3^

#### ^1^Hunter Medical Research Institute, New Lambton Heights, NSW, Australia; ^2^School of Health Sciences, The University of Newcastle, Callaghan, NSW, Australia; ^3^Hunter New England Local Health District, New Lambton, NSW, Australia

##### Correspondence: mel.hart@health.nsw.gov.au

*Journal of Eating Disorders* 2024, 12(suppl 2): O19

Background: Since the implementation of the NSW Eating Disorder Service Plan, clinical demand has increased. In response, Hunter New England Local Health District (HNELHD) has implemented models of care across metropolitan, regional, rural and remote sectors and created an innovative multidisciplinary Eating Disorders Clinical Support Team (CST) to support clinicians. In order to tailor the training and support offered to current workforce needs, a needs assessment of clinicians working in inpatient and community-based settings was conducted. Method: An online, anonymous cross-sectional survey of clinicians working in inpatient and community-based settings across all ages was conducted in November 2022 to explore education and support needs. Consultations across the LHD were conducted. Data were analysed descriptively. Results: 166 clinicians completed the survey. While most (77%) reported willingness to provide care, many did not feel confident providing assessment (51%) or treatment (45%). One-third (34%) were not confidence in detection. Clinical consultation (68%), access to eating disorder information (54%) and care navigation (48%) were identified as potentially helpful for clinicians. Access to specific training (54%), supervision (43%), group reflection (40%), mentoring (28%) and shadowing specialist clinicians (40%) were requested to support the emerging workforce. Differences across clinician groups were noted. Feedback from consultations provided further insight into the tiered requirements of clinicians, where clinical role, skill and experience varied. Conclusion: This study highlights opportunities for identifying complex clinician needs across a geographically diverse LHD, along with assisting the CST to tailor their service accordingly, to enable high value patient care and intervention.

## O20 Navigating care pathways across a geographically diverse Local Health District: defining integrated, stepped care recovery pathways within mainstream and specialist eating disorder services

### *Melissa Hart^1, 2, 3^, Julie Mavay^3^ and Warren Ward^4,5^

#### ^1^Hunter Medical Research Institute, New Lambton Heights, NSW, Australia; ^2^School of Health Sciences, The University of Newcastle, Callaghan, NSW, Australia; ^3^Hunter New England Local Health District, New Lambton, NSW, Australia; ^4^University of Queensland, St Lucia, QLD, Australia; ^5^Ramsay Clinic, New Farm, QLD, Australia

##### Correspondence: mel.hart@health.nsw.gov.au

*Journal of Eating Disorders* 2024, 12(suppl 2): O20

Background: Treatment options for people with an eating disorder are expanding, and in some instances incorporate mainstream public health services. This requires a shift in thinking about stepped care and referral options for people with an eating disorder. Despite the mortality and morbidity associated with eating disorders, difficulty in accessing care when it is needed and lack of clarity in navigating care for people with an eating disorder are known to be potential barriers to effective care. Services may also be fragmented and not meet the client at their stage of recovery. Method: A NSW Local Health District (LHD) will be used as an example of a process to define potential recovery pathways for people with an eating disorder. A scoping exercise within a LHD was conducted to explore potential services available to people with an eating disorder, treatments offered and patient eligibility criteria. Potential patient journeys at different stages of recovery were explored and mapped against existing service options. Results: A simplified stepped care model has been developed inclusive of mainstream and specialist services based on matching patient stage of recovery with the right care. Pathway decisions based are predominantly based on the psychological, nutritional and medical needs of the individual and mapping to appropriate service type. Conclusion: In order to meet the increasing demand for eating disorder treatment services at all levels of care, recovery pathways can be developed that are inclusive of mainstream and specialist services, and based on individual stage of recovery.

## O21 Initial Eating Disorder Risk Among Adolescent and Young Adults who engage in Dieting in the Community

### *Melissa Pehlivan^1^, Mirei Okada^2^, Jane Miskovic-Wheatley^1^, Sarah Barakat^1^, Stephen Touyz^1^, Stephen Simpson^2^, Andrew Holmes^2^, Sarah Maguire^1^

#### ^1^InsideOut Institute for Eating Disorders, The University of Sydney and Sydney Local Health District, Sydney, NSW, Australia; ^2^Charles Perkins Centre, The University of Sydney, Sydney, NSW, Australia

##### Correspondence: melissa.pehlivan@sydney.edu.au

*Journal of Eating Disorders* 2024, 12(suppl 2): O21

Objective: Dieting is common among young people (e.g., adolescents, young adults) and is a potent risk factor for eating disorder (ED) symptoms and development. Dieters may already carry heightened ED risk, as diets are often motivated by body image concerns, another core ED risk factor. As a step towards explaining the strong link between dieting and EDs, the current study aimed to document initial ED risk among young people.

Method: Older adolescents (> 16 years) and young adults starting or intending to start a diet (N = 421) completed a screener questionnaire assessing self-reported sociodemographic factors, past and current eating disorder symptoms, behaviours (EDE-Q) and diagnoses (including suspected but not formally diagnosed ED). Descriptive statistics were used to document ED risk among young dieters. Results: Many young dieters expressed body image concerns (43%) and a history of dieting and binge eating was common (35%). Nearly half the sample (43%) screened at-risk of an ED, with 12% likely having ED. Over 10% indicated having an ED at some point, with one third reporting experiencing symptoms, but had no formal diagnosis of an ED.

Conclusion: Many young people starting a diet may already be on a trajectory towards disordered eating or ED development. Findings suggest a high level of ED risk among young people starting a diet in the community and point to the need for greater screening and monitoring of this high-risk sample. Continued education on the challenges of dieting and encouragement of help-seeking in young people is indicated.

## O22 Exploring education and training needs for eating disorders detection and management: A survey of Australasian Dietitians

### *Munpreet Dillon^1^, Melissa Hart^2,3^, Jeremy Freeman^4^, David Sibbritt^5^, Alana Heafala^1^, Kirrilly Pursey^3^, Lauren T. Williams^1,2^

#### ^1^School of Health Sciences and Social Work, Gold Coast Campus, Griffith University, Southport, QLD, Australia; ^2^Hunter Medical Research Institute; New Lambton, NSW, Australia; ^3^School of Health Sciences, University of Newcastle, Callaghan, NSW, Australia; ^4^Australia & New Zealand Academy for Eating Disorders (ANZAED) St Leonards, NSW, Australia; ^5^School of Public Health, University of Technology, Ultimo, NSW, Australia

##### Correspondence: munpreetkdillon@gmail.com

*Journal of Eating Disorders* 2024, 12(suppl 2): O22

Background: Opportunities to advance clinical competence for dietitians working with people with an eating disorder are growing, despite limited established evidence informing needs. This study aimed to explore the perceived eating disorders education and training needs of Australasian dietitians. Method: An online cross-sectional survey (51-items) utilising closed and open-ended items was administered. Recruitment occurred through various media channels via Dietitians Australia, Australian and New Zealand Academy for Eating Disorders and InsideOut Institute for Eating Disorders. Accredited Practising Dietitians (Australia) and Registered Dietitians (New Zealand), were eligible to participate. Descriptive statistics and Pearson’s Chi-Square tests were used to analyse perceived knowledge, skill, comfort, confidence and need for education and training. Results: One hundred and forty-seven practising dietitians completed the survey. Ninety-eight percent were female (n = 144), nearly half (47%, n = 57) were aged 31–40 years and 63% (n = 93) had ≤ 5 years’ experience working as a dietitian. Although most dietitians had received eating disorders education or training, 97% (n = 143) required further training. Most did not feel skilled 56% (n = 82), or confident 62% (n = 91) in providing care to patients with an eating disorder. Dietitians' self-perception was statistically associated with years of experience, age and number of people seen with an eating disorder. Requested educational content areas reflected the complexity of training needs. Conclusion: This study highlighted a lack of perceived confidence and skill among dietitians to detect and care for people with an eating disorder. The findings provide further direction for developing tailored eating disorders education and training for dietitians at varying levels of experience.

## O23 Using trauma-informed treatments for eating disorders: Voices from a multidisciplinary roundtable

### *Phillip Aouad, Ashlea Hambleton, Erin-Jessica Laughlin, Lisa Bloom, Sue Bloom, Maree Teesson, Emma Barrett, Stephen Touyz

#### InsideOut Institute, University of Sydney, NSW, Australia

##### Correspondence: phillip.aouad@sydney.edu.au

*Journal of Eating Disorders* 2024, 12(suppl 2): O23

Taking a trauma-focused approach to eating disorder (ED) treatment allows individuals to process negative emotional memories, thoughts, and beliefs that protract the severity and length of EDs and associated comorbidities. Eye Movement Desensitization and Reprocessing (EMDR) is an emerging, evidence-based therapeutic approach that addresses recovery-interfering factors. However, several gaps need addressing before EMDR can be empirically tested in a larger ED sample. Current EMDR protocols often overlook important factors associated with eating pathology (e.g. multiple relapse-episodes) and do not serve as a 'standalone' treatment. More importantly, current ED EMDR protocols have not been informed by lived-experiences. Thus, the current study aims to address such gaps through a series of multidisciplinary round table (MDRT) discussions to evaluate existing EMDR protocols (standard and illness-specific). Clinical, academic, lived experience, carer, First Nations, LGBTQIA + , and neurodiverse perspectives were represented. The primary goal of the MDRT discussion was to ensure that the modified protocol was inclusive, sensitive, and applicable to the widest demographic possible. Emergent themes from the MDRT included an added phase of treatment (Phase 0) that provides necessary structure, direction, safety and ED specific support; an adapted Adaptive Information Processing (AIP) model for EDs (formulation vs diagnostic categories); a stronger focus on adverse impacts of biological and sociocultural influences (such as sex, sexuality, and gender; ethnicity and cultural background; neurodiversity); and, the inclusion of assessment of intergenerational trauma. Overall EMDR protocols need a shift in language and assessments that embrace inclusivity and intersectionality. Future research should work toward trialling EMDR in a diverse sample of individuals.

## O24 Digital Intervention for Body Image Dissatisfaction and Eating Disorder For Adolescents and Adults: A Narrative Review

### *Pranita Shrestha^1^, Roisin McNaney^1^, Pari Delir Haghighi^2^, Jue Xie^1^, Gemma Sharp^3^

#### ^1^Action Lab, Human-Centred Computing, Faculty of Information Technology, Monash University, Melbourne, VIC, Australia; ^2^Human-Centred Computing, Faculty of Information Technology, Monash University, Melbourne, VIC, Australia; ^3^Department of Neuroscience, Central Clinical School, Faculty of Medicine, Nursing and Health Sciences, Monash University, Melbourne, VIC, Australia

##### Correspondence: pranita.shrestha@monash.edu

*Journal of Eating Disorders* 2024, 12(suppl 2): O24

With the advancement of cutting-edge technology, various digital intervention tools have been designed and developed to provide intervention for body image dissatisfaction and eating disorders. This review aims to identify and assess the different technologies used in these digital interventions/ A systematic search strategy was conducted on 30th November 2022 on four databases namely MedLine, PsychINFO, Scopus and Web of Science, based on the PICO framework for 10 years (2012–2022). The empirical research written in English language, focusing on digital technology solutions for body image dissatisfaction and eating disorders were selected. The search retrieved 2,009 articles in total. After the removal of duplicates and the papers that fell under the exclusion criterion, 119 articles were selected. Based on the review, digital intervention technology can be divided into 5 categories: virtual reality (n = 3), web (n = 77), mobile (n = 35), chatbot (n = 2) and video games (n = 2). These interventions were either deployed in lab settings or in self-directed settings. The virtual reality (n = 3) and video games (n = 2) were fully utilised in the lab settings. Most of the web, mobile and chatbot interventions, on other hand, were utilised in a self-directed manner at home. The "one size fits all" scenario for the interventions was observed during the review of all the articles. This sizeable gap in the current literature in terms of cultural and gender inclusivity and personalisation, with regard to individual and social aspects, should be addressed in the future intervention development.

## O25 PeerED: A professional development digital learning platform for peer workers in eating disorders (*This paper is an Ignited Award Recipient)

### *Rachael Duck^1.2^, Amy Lind^1^, Dr Anita Raspovic^2.3^

#### ^1^Eating Disorders Victoria (EDV), Abbotsford, VIC, Australia; ^2^La Trobe University, Bundoora, VIC, Australia; ^3^Australian Eating Disorders Research and Translation Centre (AEDRTC), Camperdown, NSW, Australia

##### Correspondence: rachael.duck@eatingdisorders.org.au

*Journal of Eating Disorders* 2024, 12(suppl 2): O25

Background: Peer workers draw from their lived experience of being recovered from an eating disorder, to help support a person in recovery from an eating disorder in a mutually beneficial way. Ongoing professional development is integral to the conduct of peer roles and ultimately the success of this expanding workforce. However, there is a notable lack of access to eating disorder specific resources which address the unique professional development needs of this group. In 2022, Eating Disorders Victoria (EDV) secured funding through an InsideOut Institute for Eating Disorders IgnitED grant, to create a professional development digital learning platform for peer workers, titled PeerED.

Aims: The main aim of this project was to design, create and pilot PeerED, to go towards addressing the gap in ongoing professional development needs of peer workers in eating disorders. The intention is for PeerED to be further developed over time and accessible for peer workers across the eating disorder sector.

Method: A co-production process involving the expertise of five peer workers and two peer worker supervisors at EDV, was utilised to design and pilot PeerED.

Results & Conclusions: Key themes from the co-production workshops included identification of six areas of professional development needs, which were translated into PeerED modules: key concepts of peer work, sharing your story, peer supervision/support, session structure and design, information resource sharing and fostering a community of practice.

Implications/Relevance: Further piloting and evaluation is being conducted with peer workers across 2023, to optimise the efficacy, utility and relevance of PeerED.

## O26 What are the experiences of family-based therapy among adolescents affected by eating disorders? A qualitative systematic review

### *Sam Bullen^1,2,3^, Catherine Patterson^1,4,5^

#### ^1^Australian College of Nursing, Deakin, ACT, Australia; ^2^School of Nursing, Midwifery and Public Health, University of Canberra, Bruce, ACT, Australia; ^3^School of Health, University of New England, Armidale, NSW, Australia; ^4^Canberra Health Services & ACT Health, SYNERGY Nursing and Midwifery Research Centre, ACT, Australia; ^5^Robert Gordon University, Aberdeen, Scotland, UK

##### Correspondence: samantha.bullen1@health.nsw.gov.au

*Journal of Eating Disorders* 2024, 12(suppl 2): O26

Purpose: This qualitative systematic review examined the experiences of young people receiving Family Based-Therapy, to provide clinical perspectives on the topic for practice. Methods: A systematic review using meta-aggregation was conducted using the Joanna Brigg’s Institute (JBI) for qualitative evidence synthesis. The review process followed a registered review protocol and was conducted and reported based upon PRISMA guidelines. The studies were checked according to a pre-determined inclusion/exclusion criterion, and data extraction and methodological quality assessment was conducted in parallel. Results: Five publications were included. A total of 16 unequivocal and 22 credible findings and ten categories were identified, which were then synthesized into three domains. The three synthesized findings related to ‘phycological distress’, ‘relationship breakdown’ and ‘negative views of both the therapeutic alliance and the healthcare system’. Conclusion: Patient experience influenced both individual outcomes and future healthcare interactions. Adolescents articulated that their experiences where predominately negative. The gaps in the family-based therapy/ Maudsley model of care include failing to address the psychological concerns of the adolescent and focusing solely on weight restoration. Commonly, adolescents feel powerless and voiceless through this model of care. Implications for practice: Policy makers, health care professionals and researchers are very slowly making progress towards acknowledging the unique needs of adolescent living with eating disorders. Health professionals should engage in training to help meet the acute psychological distress that occurs in these settings and refer to psychological support networks including headspace and Community Adolescent Mental Health Service (CAMHS) at the first point of contact.

## O27 From evaluation to real-world implementation: Translating findings from an online self-help program for binge-eating disorder and bulimia nervosa

### *Sean Rom^1^, Sarah Barakat^1^, Stephen Touyz^1^, Sharyn Lymer^3^, Mars Kim^2^, Jessica Hazelton^1^, Morgan Sidari^1^, Matthew Fuller-Tyszkiewicz^4^, Phillip Aouad^1^, Sally Corry^1^, Jane Miskovic-Wheatley^1^, and Sarah Maguire^1^

#### ^1^The InsideOut Institute, Central Clinical School, The University of Sydney and Sydney Local Health District, Sydney, NSW, Australia; ^2^Sydney Local Health District Mental Health Services, Royal Prince Alfred Hospital, Sydney, NSW, Australia; ^3^Charles Perkins Centre, Faculty of Medicine and Health (Central Clinical School), The University of Sydney, Sydney, NSW, Australia; ^4^School of Psychology, Deakin University, Geelong, VIC, Australia

##### Correspondence: sean.rom@sydney.edu.au

*Journal of Eating Disorders* 2024, 12(suppl 2): O27

Binge Eating eTherapy (BEeT) is one of the first online CBT programs for eating disorders (ED) in Australia. BEeT consists of interactive, multi-media content and inbuilt digital calendar with self-monitoring tools. This presentation will deliver key findings from previous and ongoing evaluations of BEeT, including a recently completed randomised controlled trial (RCT) in those with full or sub-threshold bulimia nervosa (BN; n = 114), a pilot study in women with binge-eating disorder (BED; n = 19), and an ongoing real-world implementation study in collaboration with headspace (n to date = 24). Findings from both the RCT and the BED pilot study demonstrated very promising outcomes. Both clinician-supported and pure self-help treatment arms were equally effective in reducing ED psychopathology from baseline to post-treatment (compared with control) in the RCT, whilst the BED pilot study provided additional support for clinician-supported BEeT with large, statistically significant reductions in OBE frequency (b = -9.84, p < 0.001, g = 1.03) and ED psychopathology (b = -1.83, p < 0.001, g = 1.62) from baseline to post-treatment, maintained at follow-up. Taken together, these findings suggest that low-resource interventions such as online self-help programs may increase accessibility to effective, evidence-based care for individuals with a non-underweight ED. An overview of our progress, in collaboration with headspace, to provide the BEeT intervention to young people (12–25 yrs) experiencing mild to moderate symptoms of binge-eating or compensatory behaviour will also be discussed, including the challenges involved in translating findings from rigidly controlled studies to real-world contexts.

## O28 A national survey to inform a more comprehensive understanding and support of parents with a child experiencing an eating disorder

### *Simon Wilksch^1,2^

#### ^1^College of Education, Psychology and Social Work, Flinders University, Bedford Park, SA, Australia; ^2^Advanced Psychology Services, Adelaide, SA, Australia

##### Correspondence: simon.wilksch@flinders.edu.au

*Journal of Eating Disorders* 2024, 12(suppl 2): O28

LINK—Toward a more comprehensive understanding and support of parents with a child experiencing an eating disorder—Wilksch—2023—International Journal of Eating Disorders—Wiley Online Library. https://onlinelibrary.wiley.com/doi/10.1002/eat.23938.

## O29 Alexithymia as a Transdiagnostic Factor across Depressive, Anxiety and Eating Disorders in Adults: A *Meta*-Analytic Review

### Sophie Rachel Yeung^1^, Litza Kiropoulos^1^, An Dang^1^, Matthew Fuller-Tyszkiewicz^2^, Chi Nguyen^1^, Edric Stephanus Widjaja^1^, *Isabel Krug^1^

#### ^1^The University of Melbourne, Melbourne, VIC, Australia; ^2^Deakin University, Geelong, VIC, Australia

##### Correspondence: isabel.krug@unimelb.edu.au

*Journal of Eating Disorders* 2024, 12(suppl 2): O29

Depressive disorders (DDs), anxiety disorders (ADs) and eating disorders (EDs) are highly prevalent and often comorbid disorders. Thus, there has been growing interest in investigating transdiagnostic factors such as alexithymia, which refers to difficulties identifying feelings (DIF), difficulties describing feelings (DDF) and externally-oriented thinking (EOT) and is most widely measured using the Toronto Alexithymia Scale (TAS). This meta-analysis aimed to synthesise the literature comparing TAS total and subscale (i.e., DIF, DDF and EOT) scores between healthy controls and DD, AD and ED adult populations and to summarise the relationship between DD, AD and ED symptomatology and TAS scores. Electronic databases, PsycInfo, Embase and Medline, were systematically searched. Sixty-eight studies were included in the meta-analyses which were separated into: DD, AD and ED groups and specific disorders within these groups where possible. Random-effects modelling was used for both group differences and correlations where standardised mean differences and Pearson correlations were used for effect sizes respectively. TAS total, DIF and DDF scores were significantly higher across DD, AD and ED groups (more specifically, across major depressive disorder, anorexia nervosa, bulimia nervosa and panic disorder) compared to healthy controls. Significant positive associations between disorder symptom severity and TAS, DIF and DDF scores were found in all mental health groups (specifically, in major depressive disorder, anorexia nervosa and panic disorder). In conclusion, alexithymia may be both trans-diagnostically elevated across and related to symptom severity in DDs, ADs and EDs, indicating assessing alexithymia in these populations and developing effective transdiagnostic treatments are warranted.

## O30 Psilocybin assisted psychotherapy for treatment-resistant Anorexia nervosa

### *Stephen Touyz^1^, Sarah Maguire^1^, Kristi Griffiths^1^

#### ^1^InsideOut Institute for Eating Disorders, Faculty of Medicine and Health, University of Sydney, NSW, Australia

##### Correspondence: stephen.touyz@sydney.edu.au

*Journal of Eating Disorders* 2024, 12(suppl 2): O30

In a recent Journal of Eating Disorders editorial, Touyz and Hay (2022) argued for greater innovation in the field of eating disorders, calling for urgent paradigm shifts. Whilst many have benefited from existing evidenced-based therapeutics, research shows that up to 40% of individuals with anorexia nervosa (AN) will still be ill at 20 years. AN maintains the highest mortality rate of the mental disorders and is the leading cause of mental health related hospitalisations in Australia. The high mortality rate and costs of AN reflect a conspicuous lack of treatments, as there are no recognised or effective pharmacotherapies for AN. Preliminary trials have shown notable therapeutic potential in a range of severe refractory psychiatric illnesses following 2–3 spaced exposures to psilocybin embedded within a carefully managed psychotherapeutic regime, so called psilocybin-assisted psychotherapy. Ambivalence, cognitive rigidity and amotivation are central to AN and are important predictors of treatment response and relapse prevention. Participants given psilocybin report lasting increases in open-mindedness and psychological/cognitive flexibility, readiness to try and engage in new activities, and improved ability to dismantle rigid and habitual mental templates in several psychiatric disorders that are often co-morbid with AN. Psilocybin-assisted psychotherapy in AN is being trialled in the USA, UK and Australia. There is emerging evidence of a biological candidate marker for AN (emotion elicited ERP) and brain circuitry dysregulation may be the root cause of this devasting disorder. This paper will address the potential for mapping the neuro-biotypes for AN and how they may influence clinical outcomes. |

## O31 Dietitian's confidence and practice in the management of eating disorders during pregnancy

### *Tamara Parker^1^, Rebecca Angus^1^

#### ^1^Allied Health and Rehabilitation Services, Gold Coast Hospital and Health Service, Gold Coast, QLD, Australia

##### Correspondence: tamara@nourishingdietetics.com.au

*Journal of Eating Disorders* 2024, 12(suppl 2): O31

Background: Dietitians who care for patients with eating disorders (EDs) are expected to have undergone advanced training to develop knowledge and skills according to the “ANZAED practice and training standards for dietitians providing ED treatment”. Studies have found dietitians lack confidence and self-reported skill in treating patients with an ED. There are no studies looking at confidence or practice of dietitians working with people with EDs during pregnancy. As EDs are estimated to affect 5.2–7.5% of pregnant people it is imperative that dietitians possess the skill, knowledge, and confidence to treat this population.

Aim: To assess current practice, confidence, and training needs of dietitians treating EDs in pregnancy.

Methods: A cross-sectional survey of Australian dietitians with past year experience in ED, antenatal or both fields was completed using MS Forms between Nov 2022 – Jan 2023.

Results: 117 responses were analysed. Time as a dietitian, but not recent experience in the population increased confidence to assess a person with an ED in pregnancy (P < 0.01). Dietitians were more likely to weigh a person with an ED in pregnancy, however, were less likely to provide weight or kilojoule targets. The majority indicated further training (93%) and guidelines (98%) would be helpful.

Importance: This is the first study assessing dietitians’ current practice and confidence in screening, assessing and treating EDs during pregnancy. It highlights low levels of confidence, conflicting interventions provided, variation from current available guidelines for ED and antenatal populations, and the overwhelming need for access to training and guidelines.

## O32 Eating Disorders *Victoria*'s Severe and Enduring Eating Disorders community program—a lived experience co-design and delivery program

### *Zoe Sweeney^1^, Richard Knight^1^, Rebecca Lister^1^

#### ^1^Eating Disorders Victoria, Abbortsford, VIC, Australia

##### Correspondence: zoe.sweeney@eatingdisorders.org.au

*Journal of Eating Disorders* 2024, 12(suppl 2): O32

Eating Disorders Victoria’s (EDV) development of a co-designed, community-based program for people with Severe and Enduring Eating Disorders (SE-ED). Background: SE-ED are intensive illnesses, however effective evidence-based treatments are limited. In response, EDV developed the SE-ED pilot program.

Aims: Describe the co-design processes used in the development of the EDV SE-ED program and outline program results.

Methods: A variety of methods were used to gain feedback from consumers with lived experience of SE-ED for the development of the program: 118 consumers provided qualitative feedback about living with a SE-ED; 20 consumers participated in a two-hour online focus group and an advisory group, made up of both lived experience participants and other professionals Four models of practice where utilised within the program design: psychosocial and educational group work; group peer mentoring; one-on-one wellbeing check-ins and social engagement. Lived experience staff guided participants to learn and re-engage in meaningful roles and activities and implement new skills. Results: Four rounds of the EDV SE-ED program have now been delivered. 58 participants completed the program with approximately 80% recording an improvement in their Quality of Life. An increase in help- seeking behaviour, higher levels of engagement with existing care team and engaging new supports was also noted. Key findings: Co-design is central to the success of the pilot; lived experience staff are critical change agents; focus on improving quality of life rather than recovery; relationships, based on collective understanding of shared experience, self-determination and empowerment and the program holds potential for future testing.

## P1 Screening to Capture Nutritional Complexity and Parental Knowledge/Confidence with Feeding in Eating Disorder Treatment at the Child and Youth Mental Health Service Eating Disorder Program (CYMHS EDP)

### *Amy Davis^1^, Grace Conforti^2^, Gabryela Sellars^2^, Morgan Sidari^1^, Dr Helen MacLaughlin^2^

#### ^1^Queensland Health, Brisbane, QLD, Australia; ^2^Queensland University of Technology, Brisbane, QLD, Australia

##### Correspondence: Amy.Davis@health.qld.gov.au

*Journal of Eating Disorders* 2024, 12(suppl 2): P1

Young people (YP) presenting for eating disorder treatment differ in their degree of nutritional complexity, which could contribute to inequity in treatment outcomes. Parental feeding knowledge and confidence will also differ and has the potential to impact treatment. We developed a Parent Nutrition Screening Tool (PNST) with the aim to identify families with complex nutritional presentations (eg. food allergies) and parents with low knowledge/confidence with feeding. The PNST was completed by consenting parents of 112 YP upon initial presentation at CYMHS EDP: a specialist eating disorder service for YP up to the age of 18 with a diagnosed eating disorder in the Greater Brisbane Area. The tool contained eight items relating to the presence and nature of nutritional complexities and two items relating to parental feeding knowledge and confidence. The PNST identified that 83% of YP are presenting with ≥ 1 nutritional complexity and 14% of families rate their feeding knowledge and/or confidence in the lowest category. This high response rate indicates that the majority of the cohort may benefit from adjunct dietetic support. I will discuss an overview of the nutritional complexities in our cohort, as captured by the PNST, along with the parental feeding skill and confidence measures and their relationship with parental self-efficacy. These analyses are being used to inform a revised version of the tool, which we hope will capture those families with the highest need that may benefit from early nutrition assessment and support from a dietetic professional in our resource restricted setting.

## P2 Implementation of an eating disorder screening and care pathway in a general mental health inpatient service

### *Amy Kaplan^1^, Suzie Hooper^1^, Ana Hutchinson^2,3^, Karen Gwee^1^, Lola Valent^1^, Damien Khaw^2,3^, Jane Willcox^2,3^

#### ^1^Epworth Clinic, Epworth HealthCare, Camberwell, VIC, Australia; ^2^Centre for Quality and Patient Safety Research, Epworth HealthCare/Deakin University HealthCare Partnership, Richmond, VIC, Australia; ^3^Institute for Health Transformation, Deakin University, Burwood, NSW, Australia

##### Correspondence: amy.kaplan@epworth.org.au

*Journal of Eating Disorders* 2024, 12(suppl 2): P2

Aim: General mental health (MH) inpatient units hold a valuable place in the stepped system of treatment options for consumers with eating disorders (ED) or disordered eating (DE). This study aimed to implement a standardised evidence-based screening and care pathway to improve identification and treatment of inpatients with ED/DE’s on a general MH ward.

Methods: Informed by implementation science frameworks, pre-admission screening (SCOFF tool) was embedded into practice, a multi-disciplinary evidence-based care pathway was developed and implemented, supported by a health professional (HP) ED/DE education program.

Results: Process and implementation data were compared for three-month periods pre (2019, n = 348) and post-implementation (2021, n = 284). Post-implementation, SCOFF screening occurred in 94.7% of admissions and scores ≥ 2 were significantly predictive of ED/DE (OR = 35.2, p < 0.001). The odds of identifying significantly DE (potentially undiagnosed ED) were 3.1 times greater (p = 0.003). Post-implementation, for those with an ED/DE, dietitian referrals were 2.5 times more likely (p < 0.001), and provision of a documented management plan 2.7 times greater (p = 0.044). 50 HP’s undertook tailored ED and meal support therapy training. Pre and post training evaluation showed improvements in feeling supported in this clinical area (45% vs 79%) and application of meal support therapy skills (61% vs 86%).

Discussion: An ED/DE screening and care pathway, along with HP training, can be feasibly implemented in a general MH inpatient setting. This process can identify more consumers at risk of ED/DE, allowing them to obtain consistent evidenced based care during admission.

## P3 The Australian Eating Disorder Workforce Research Survey: What does the evidence say about our research engagement and capacity?

### *Anita Raspovic^1,2^, Clare Foldi^3,4^, Siân McLean^5^, Felicia Reed^2,3,4^, Hugh Bidstrup^1,6^, Leah Brennan^1^

#### ^1^School of Psychology and Public Health, La Trobe University, Wodonga, VIC, Australia; ^2^Australian Eating Disorders Research and Translation Centre (AEDRTC), NSW, Australia; ^3^Department of Physiology, Monash University Clayton, VIC, Australia; ^4^Monash Biomedicine Discovery Institute, Monash University Clayton, VIC, Australia; ^5^School of Psychology and Public Health, La Trobe University, Melbourne, VIC, Australia; ^6^School of Behavioural and Health Sciences, Australian Catholic University, Melbourne, VIC, Australia

##### Correspondence: a.raspovic@latrobe.edu.au

*Journal of Eating Disorders* 2024, 12(suppl 2): P3

Background: Increasing research engagement and capacity within the Australian eating disorder academic, clinical and lived experience workforce is a key workstream of the Australian Eating Disorders Research and Translation Centre (AEDRTC). The impetus for workforce research engagement and capacity development is multi-fold, including worker, service and societal level benefits. Ultimately however, more successful translation and implementation of research across this workforce aims to improve outcomes for those experiencing eating disorders and their loved ones. Our current understanding of research engagement and capacity across the diverse Australian eating disorder workforce, and how to optimise its uptake, is limited. Scoping work is required to tailor frameworks to building research engagement and capacity across the sector.

Aims: To determine research engagement and capacity, and identify research support and training needs, of the Australian eating disorder workforce.

Methods: A 5-year online survey of the Australian eating disorder workforce commenced in 2023. The survey is both cross-sectional and prospective/longitudinal in design and will be re-administered annually. Existing workforce research engagement measures are administered via branching logic, where respondents are given question sets relevant to their roles and level of research engagement.

Results and Conclusions: Initial results will be available in 2023 / 2024.

Funding acknowledgement: This study is an activity of the Australian Eating Disorders Research and Translation Centre which is funded by the Australian Government Department of Health and Aging under the National Leadership in Mental Health Program.

## P4 The Road to TEDS: Establishing a Specialist Eating Disorder Service for Tasmania

### *Anita Reimann^1^, Meg Boman^1^, Lara McCartney^1^, Jess Oakford^1^, Kristina McDonnell^1^

#### ^1^Tasmanian Health Service. Department of Health, Hobart, TAS, Australia

##### Correspondence: anita.reimann@health.tas.gov.au

*Journal of Eating Disorders* 2024, 12(suppl 2): P4

There is currently no statewide specialist adult eating disorder service in Tasmania. Services are available for children and young people through Child and Adolescent Mental Health and paediatric services. The National Eating Disorder Collaboration (NEDC) Stepped Model of Care Framework outlines the components of a stepped system of care. Tasmania is missing two essential components of the complete system- Community-based Intensive Treatment and a Residential Treatment Program. The Tasmanian Eating Disorder Service (TEDS) is being established by the Tasmanian Department of Health. The Australian Government have provided funding to plan and design the model of care, including infrastructure needs, for the establishment of specialist eating disorder services. It will take more than a village working together to establish TEDS. How will TEDS bridge the current gaps and create a system or ‘village of care’ that can meet the needs of people with an eating disorder, and deliver a sustainable and effective service? In this presentation we will detail lived experience, consumer perspectives of service gaps and the types of support and interventions needed, including service navigation and access. Sector and system issues identified by stakeholders, such as workforce capacity and capability to deliver specialist interventions and support will also be explored. We will describe the proposed TEDS model of care which has been informed by a co-design methodology which has been embedded through the service development process. The importance of meaningful engagement of stakeholders and how this underpins the effective connection and integration of TEDS with existing service will also be discussed.

## P5 Butterfly: Let’s Talk – a podcast providing information and hope for recovery for anyone affected by body image issues or an eating disorder

### *Camilla Becket^1^, Sam Ikin^2^, Melissa Wilton^1^

#### ^1^Butterfly Foundation, Crows Nest, NSW, Australia; ^2^Ikin Media, Kensington, TAS, Australia

##### Correspondence: camilla.becket@butterfly.org.au

*Journal of Eating Disorders* 2024, 12(suppl 2): P5

Introduction: The Butterfly podcast connects listeners with experts and people with lived experience of an eating disorder and/or a body image concern. In an exploding market for podcasts, Let’s Talk builds public awareness around the issues; showcases professional knowledge; disseminates research, and provides much needed hope for recovery.

The Need: Research shows a lack of public awareness and understanding underlies pervasive minimisation, stigma, and stereotyping around eating disorders. This leads to cycles of shame, secrecy, and symptoms for people affected. Let’s Talk provides a space for conversation, because talking helps.

Objectives: Address common myths and stereotypes and other barriers to help seeking; Showcase diversity of those affected; Decrease shame and stigma; Increase awareness of signs, symptoms, interventions and supports; Build awareness of diverse sociocultural contexts; Shift the culture towards greater acceptance of diversity in weight, size, colour, and shape.

Stats & Facts: Launched in June 2020, Let’s Talk is currently in its third year. To date we have produced 31 episodes with 77,327 total downloads, equalling an average of 2,494 downloads per episode with more than 650 listens per week. The podcast has also received the following: New and Noteworthy Apple podcast 2020; Mental Health Service Award Winner 2021; Shortlisted: 2021 Tasmanian Media Awards Public Service Journalism. According to Buzzsprout, Let’s Talk is in the top 10% of podcast downloads worldwide.

Analysis: This poster presentation will review Butterfly’s rationale, objectives, and development of the podcast with key learnings around format, content, demographics, promotion, reach, engagement, and feedback from consumers.

## P6 EveryBODY Welcome! The launch of the National Collaboration for diverse and inclusive body image and eating disorders supports

### *Annaleise Robertson^1^, Mandy Goldstein^1^, Peta Marks^2^, Melissa Wilton^3^, Jennifer Paterson^4^, Laurence Cobbaert^5^, Jane Miskovic-Wheatley^6^, Beth Shelton^7^

#### ^1^Australia and New Zealand Academy for Eating Disorders, St Leonards, NSW, Australia; ^2^Australian Eating Disorder Research and Translation Centre, NSW, Australia; ^3^Butterfly, Crows Nest, NSW, Australia; ^4^Eating Disorders Families Australia, Parkville, VIC, Australia; ^5^Eating Disorders Neurodiversity Australia, NSW, Australia; ^6^InsideOut Institute for Eating Disorders, Camperdown, NSW, Australia; ^7^National Eating Disorders Collaboration, Crows Nest, NSW, Australia

##### Correspondence: annaleise.robertson@health.nsw.gov.au

*Journal of Eating Disorders* 2024, 12(suppl 2): P6

Eating disorders are serious mental illnesses associated with high rates of mortality and low rates of detection and intervention. They disproportionately affect people who identify as LGBTQIA + who also face unique barriers to care. The everyBODYwelcome! (eBw!) initiative, a first-time collaboration of Australia’s seven national eating disorder organisations, launched as part of the 2023 Sydney World Pride celebrations to raise awareness, reduce stigma and commit to advocacy and improvements in healthcare delivery for diverse populations. The eBw! collaboration met monthly for 6 months to identify need from each organisation’s perspective, engaged in a brief literature and service review, and consulted with people with lived experience from the LGBTQIA + community to design key messaging, create resources and set strategic direction.In February, 2023, eBw! presented a stall at Fair Day, the largest community event on the Mardi Gras calendar with over 80,000 people in attendance. Over 30 volunteers from the organisations assisted with the stall, 2000 items (keyrings and buttons) with a QR code to a link tree to the organisations and resources were distributed, and two national media outlets covered the launch. The volunteers on the stall received a very enthusiastic reception, with many visitors exclaiming how crucial it was to be represented at such an event. Some people even spoke about their ED experience for the very first time. Next steps for the eBw! collaboration will be to welcome broad participation from within the ED field, prepare for 2024 events, and create specific LGBTQIA + studies and co-design support guidelines.

## P7 Mingling with eating disorders: Feasibility of an online RO DBT program for people with and without eating disorders

### *Hoiyan Karen Li^1^

#### ^1^Couragepsyc, Robina, QLD, Australia

##### Correspondence: karen@drkarenli.com.au

*Journal of Eating Disorders* 2024, **12**(**suppl 2**): P7

While there are benefits of running group based treatments for people with eating disorders, there are known risks associated with group eating disorders treatment. Radically Open Dialectical Therapy (RO DBT) is a relatively newcomer in the treatment of eating disorders (Lynch et al., 2013; Isaksson et al., 2021; Baudinet et al., 2021). Instead of targeting individual diagnoses, RO DBT aims to treat underlying maladaptive overcontrol and loneliness. There is little known about transdiagnostic group outcomes for RO DBT sessions. The current study highlights the modification of the standardized RO DBT treatment program to a 24 classes × 1.5 h online group-based program with rolling intake every 6 sessions. Data is presented from 2 years of group sessions in a private practice with 15 intake sessions and 36 participants represented (average age 21.87). 38% (n = 14) of the participants reported the presence of an eating disorder. Participants with eating disorders presented at various stages of eating disorders treatment (assessment phase through to relapse prevention and remission). Preliminary qualitative feedback revealed positive outcomes on overall quality of life as well as improvements in regular eating for participants with eating disorders even though eating disorders were not specifically targeted in treatment. This brief study shows promise of treating underlying transdiagnostic overcontrol as an adjunct to standard eating disorders treatment. Further research is required, in particular, larger scale trials of RO DBT for eating disorders.

## P8 Embedding the ANZAED Practice & Training Standards to support consistency and quality across training specific to eating disorders

### *Sarah Trobe^1^, Kim Hurst^2,3^

#### ^1^NEDC, Crows Nest, NSW, Australia; ^2^Robina Private, Robina, QLD, Australia; ^3^ANZAED, St Leonard, NSW, Australia

##### Correspondence: sarah.trobe@nedc.com.au

*Journal of Eating Disorders* 2024, 12(suppl 2): P8

Health professionals require training that matches their role and equips them to provide support for people experiencing an eating disorder. High-quality training programs exist within Australia, however these have frequently differed in course content, delivery format, duration, cost, and availability. To help address this gap, The National Framework for Eating Disorders Training – A guide for training providers (the Framework) was developed. Its aim is to ensure consistency and quality across trainings and provide clear professional development pathways for mental health professionals and dietitians. A process evaluation is described, including key components of the Framework.

Method: The Framework synthesises the NEDC Workforce Core Competencies and ANZAED Clinical Practice and Training Standards to detail the knowledge and skills required for health professionals working with people at risk of or are experiencing an eating disorder. The Framework was refined through consultation with training providers from across Australia, and a process for assessing training based on the Framework was developed.

Results: The Framework development process will be presented, and overview of the agreed standards provided. Data regarding the training approvals process will be provided as a practical example of the active implementation of documented standards in workforce development initiatives.

Conclusion: The Framework guides the development of training which engages, inspires, and equips the workforce with the knowledge and skill to safely and effectively respond to, and provide treatment for people experiencing an eating disorder. It can be utilised across workforce activities at scale, supporting the growth of a skilled and competent workforce.

## P9 What can we learn from and understand about supervisors and mentors to drive improvement and support of the eating disorder workforce in Australia?

### *Emma Spiel^1^, Sarah Trobe^1^, Beth Shelton^1^, Gabriella Heruc^2^, Kim Hurst^2,4^, Siân McLean^2,3^

#### ^1^NEDC, Crows Nest, NSW, Australia; ^2^ANZAED, St Leonards, NSW, Australia; ^3^La Trobe University, Bundoora, VIC, Australia; ^4^Robina Private, Robina, QLD, Australia

##### Correspondence: emma.spiel@nedc.com.au

*Journal of Eating Disorders* 2024, 12(suppl 2): P9

A skilled and thriving eating disorders (ED) workforce requires the support and guidance of senior clinicians, mentors, and supervisors. Evidence suggests that supervision contributes to improved clinician skill and confidence, and improved outcomes for the people accessing their services, especially when evidence-based approaches are used. However, the characteristics and perspectives of and approaches used by the ED supervisory workforce within Australia are largely unknown. This research aimed to address this gap.

Methods: Participants include up to n = 50 mental health professionals and dietitians who provided supervision as part of a funded professional development program of training and supervision as part of the ANZAED Eating Disorder Credential system. Survey data was collected following delivery of all supervision sessions. It assesses supervisor demographic information, perspectives on workforce needs, and approaches used in supervision.

Results: Demographic data will be presented that articulates the experience, main areas of practice and service offerings of supervisors, noting gaps and strengths in coverage relative to workforce needs. A summary of the evidence-based approaches used in supervision, and supervisor perspectives on the professional development needs of mental health clinicians and dietitians will be presented.

Conclusions: Perspectives on the strengths and gaps of both the ED clinician and supervisor workforces and the implications of these for broader ED workforce initiatives will be discussed.

## P10 Disordered eating and diabetes: a systematic narrative review of intervention studies

### *Emma Reid^1^, Assoc. Prof. Talitha Best^1^, Dr. Alicia Carter^1^ and Dr Melissa Oxlad^2^

#### ^1^Central Queensland University, Brisbane, QLD, Australia; ^2^University of Adelaide, South Australia, Australia

##### Correspondence: emma.k.reid@cqumail.com

*Journal of Eating Disorders* 2024, 12(suppl 2): P10

Disordered eating is highly prevalent amongst individuals living with diabetes, with rates up to 39.3% in adolescents with Type 1 Diabetes (T1DM) and up to 40% in individuals with Type 2 Diabetes (T2DM). Eating disorders are associated with worse diabetes outcomes, including higher average blood glucose levels, higher rates of diabetic ketoacidosis and hospitalisation, increased diabetes complications, and higher rates of depression and anxiety. However, there is limited research or clinical guidance on interventions to address this comorbidity. A systematic narrative review was conducted to identify existing evidence-based interventions. Electronic databases PubMed, Embase and PsycINFO were searched, and 17 articles were subsequently reviewed. Interventions fell broadly into the categories of inpatient and residential (n = 3), pharmacological (n = 1), weight loss focused (n = 3), psychoeducational (n = 3), and psychological (n = 8). Cognitive-behavioural therapy was most common (n = 4), and appears to improve psychological outcomes in those living with T2DM. More intensive interventions may be required for individuals living with T1DM, with inpatient treatment found to be significantly more effective than outpatient treatment. However, even with intensive treatment, people living with T1DM were less likely to achieve eating disorder remission than peers without diabetes. Significant methodological constraints were identified amongst intervention trials including lack of randomisation or control group, small sample sizes when focused on disordered eating, limited follow-up, and largely homogenous participants involving predominantly females from Western backgrounds. This clinical review highlights the significant limitations in our knowledge of the treatment of disordered eating in diabetes. Significantly more research is needed to develop and validate tailored interventions.

## P11 The Impact of COVID-19 on Eating Disorders: A Systematic Review Examining Changes to Emergency Room Admissions

### *Grace Collinson^1^

#### ^1^Australian College of Applied Psychology, Sydney, NSW, Australia

##### Correspondence: grace_tambree@hotmail.com

*Journal of Eating Disorders* 2024, 12(suppl 2): P11

Individuals with a pre-existing mental health diagnosis, have been identified as being particularly vulnerable to the impacts of pandemics. Individuals with eating disorders appear to be at significant risk when navigating the recovery trajectory alongside the COVID-19 pandemic, because of specific vulnerabilities, such as, changes in routine, restricted access to supports, a reduction in treatment options, and increased exposure to news and social medica coverage. Research suggests that the COVID-19 pandemic may impact the relationship between the illness severity of eating disorders, and the subsequent need for admissions to hospital. Due to the recent and fast evolving nature of COVID-19, the impact of the COVID-19 pandemic is yet to be fully established. As such, we sought to systematically examine the impact of the COVID-19 pandemic on eating disorder presentations to emergency rooms. We hypothesised that the rate of eating disorder related presentations to emergency departments would increase following the COVID-19 pandemic. We analysed data collected from nine papers, recording eating disorder patient admission numbers to emergency departments across the globe between 2018 to 2022. Throughout all papers, we found that there was trend level data showing a significant increase in admissions to emergency departments for individuals with eating disorders following the onset of the COVID-19 pandemic. Interestingly we also noted a trend in more severe markers of illness severity across papers examining the implications of COVID-19 amongst children and adolescents with eating disorders. This research has the potential to inform the allocation of mental health resources, and eating disorder treatment reforms moving forward.

## P12 Investigating a reduction in *naso*-gastric tube feeding under restraint and co-designing dining room support in a specialist eating disorder unit.

### *Helen West^1,2^, Oli Williams^1^, Glenn Robert^1^

#### ^1^King’s College London, London, United Kingdom; ^2^Elysium Healthcare, Hertfordshire, United Kingdom

##### Correspondence: helen.west@kcl.ac.uk

*Journal of Eating Disorders* 2024, 12(suppl 2): P12

This research developed from a quality improvement (QI) project designed to reduce naso-gastric tube (NGT) feeding under restraint at a 16-bed inpatient eating disorder unit for 12-18yr olds. Staff had raised concerns that routine delivery of NGT feeding under restraint was having iatrogenic effects. In response, a QI process led to revising the unit’s feeding policy in line with dietetic best practice guidance. Implemented in June 2021, the new policy drastically reduced NGT feeding under restraint from a pre-intervention high of 56 incidences in one month, to 2 incidences in 6 months. While this demonstrates a significant rate of change, the impacts are not yet fully understood. In this clinical PhD fellowship, data collected via quantitative (service and patient records) and qualitative (ethnographic observation and interviews) methods will be used to examine how this policy change impacted patients and staff. Findings will be used to design guidelines that: (1) support replicable and scalable improvement (2) address gaps in existing guidance. The interrelated issue of dining room support will also be addressed. If restrained feeding is to be limited, effective alternatives for ensuring inpatients meet dietary needs are needed. Experience-based Co-Design (EBCD) will be used to bring together unit staff, researchers, parents/carers, and patients to co-design new policy and guidance for dining room support. The EBCD process will be completed in parallel by two patient groups—(1) current inpatients (2) former inpatients progressing well in their recovery – to explore whether/how state of illness/recovery influences contributions to the co-design process.

## P13 A Resource for Supporting Clinicians in Managing Adolescent Violence in the Context of Treatment for Eating Disorders

### *Jessica Ryan^1^, Gillian Cassar^1^, Bliss Jackman^1^, Alexandra Griffin^1^

#### ^1^The Victorian Centre of Excellence in Eating Disorders, Parkville, VIC, Australia

##### Correspondence: Jessica.Ryan@mh.org.au

*Journal of Eating Disorders* 2024, 12(suppl 2): P13

Young people living with anorexia nervosa (AN) commonly experience personality and temperament traits such as anxiety, which can become exacerbated as a result of the development of AN (Hill, Knatz Peck & Wierenga, 2022; Kaye et al., 2015). Eating disorder treatments, such as Family Based Therapy (FBT), can lead to a temporary increase in distress for the adolescent early in treatment. It is not uncommon for this distress to be expressed through uncharacteristic violence or aggression. Experience in practice, and discussion with public-sector services, suggest that adolescent violence is an increasing concern for families and clinicians participating in eating disorder treatment (Aradas et al., 2019). Families often experience shame about the emergence of violence, leading to a sense of isolation, increased risk in the home, as well as hopelessness and low parental self-efficacy (Shimoshoni et al., 2021). While clinicians, often feel unclear on how to support the family system (Rutter et al., 2022). This is a concern for researchers, policy makers and service providers, making this issue a key priority as a public health initiative. In response, The Victorian Centre of Excellence in Eating Disorders (CEED) has developed a preliminary resource to guide clinicians in supporting families in responding adolescent violence in the context of FBT. This resource is informed by lived experience and developed in consultation with service providers at both a state and national level. This resource aims to increase clinician competence and confidence to work with families in effectively responding to adolescent violence.

## P14 Diagrammatic navigation of the dynamics of eating disorders

### *Jolyon Grimwade^1,2^

#### ^1^Change Consultations, Essendon, VIC, Australia; ^2^First Peoples' Health and Wellbeing, Thomastown, VIC, Australia

##### Correspondence: jolyongrimwade@gmail.com

*Journal of Eating Disorders* 2024, 12(suppl 2): P14

Eating disorders are diverse but have overlapping issues and themes. It is proposed that a large table can provide a guide to thoughtful interrogation of the particular challenges of moving from preadolescence toward a version of adulthood that is satisfying. The move to adulthood is primarily shaped by a rejection, partial if not total, of each of the parents as versions of appropriate adulthood. There are eight conceptual frames for the challenges: parental identification, other identification, nurturance, food concept, body concept, persistence, life narrative, and parental discord. Each of these frames has a triangular set of descriptors with a healthy version and two, usually opposed, unhealthy preferences. These eight triangles describe different eating disorders and suggest interventional strategies. There is a ninth triangle: the triangle of personhood: me (subjectivity), myself (objectiveness), and I (agency). As adults, these aspects of person work in complement and in parallel. In the concrete experience of early adolescence the aspects of personhood do not easily coalesce; which creates for confusion in the developing pre-adult. The young person will resolve the confusion in favour of some aspect of food, body, life narrative, parental discord, parental persistence, parental nurturance, and identification with parents and others as eight conceptual frames. The poster provides diagrammatic representation of the table and the triangles.

## P15 Exploring stakeholders’ views of a new eating disorder care pathway and education program in a general mental health ward

### Jane Willcox^1,2^, Suzie Hooper^3^, Ana Hutchinson^1,2^, Karen Gwee^3^, Lola Valent^3^, *Amy Kaplan^3^

#### ^1^Centre for Quality and Patient Safety Research, Epworth HealthCare/ Deakin University HealthCare Partnership, Richmond, VIC, Australia; ^2^Institute for Health Transformation, Deakin University, Burwood, NSW, Australia; ^3^Epworth Clinic, Epworth HealthCare, Camberwell, VIC, Australia

##### Correspondence: amy.kaplan@epworth.org.au

*Journal of Eating Disorders* 2024, 12(suppl 2): P15

Aim: Effective implementation engages stakeholders, and encourages support for program processes. This study aimed to explore inpatient consumer and health professional (HP) stakeholder experience with a new eating disorder (ED) screening and care pathway and education program in a general mental health (MH) inpatient service.

Methods: Descriptive qualitative methodology utilised individual interviews and focus groups with 7 consumers and 18 HPs (mean 7.8 years MH experience). Questions included knowledge and experience of the ED care pathway, facilitators and barriers, and perceptions of person-centred care. Transcriptions were thematically analysed.

Results: Four key themes emerged.

1. Identification of ED through screening is critical and enables instigation of care often missed in the general MH environment. “The fact that the dietitian came to me. For example during [other facility] stays ….I didn’t eat for the month I was in there. Um, and nobody picked up on it.”

2. Implementation of evidence-based best practice care that had not been provided previously. “I had the right support in the right place at the right time.”

3. Extensive improvement in clinician knowledge and confidence, achieved through HP and care pathway training.

4. Recognition that embedding new care practice was seen to be an evolution and a “journey”.

Discussion: Understanding stakeholders’ experiences of new care practices enables the identification of enablers and barriers for implementation, and avenues to optimise care for consumers with ED’s in the general MH setting.

## P16 Art Therapy—a novel contributor in enhanced integrated care—supporting distress reduction during medical admission

### *Kate Morris^1^

#### ^1^Royal Melbourne Hospital—Specialist Unit: Eating Disorders, Parkville, VIC, Australia

##### Correspondence: kate.morris2@mh.org.au

*Journal of Eating Disorders* 2024, 12(suppl 2): P16

In July 2021 an Art Therapist was recruited to join the newly established Enhanced Integrated Model.

for Eating Disorders at the Royal Melbourne Hospital. A small team was formed, including a senior mental health nurse, an art therapist and a carer peer support worker. This team has provided bedside care and carer liaison to eating disorder patients and their families for the past two years. Art therapy has been an innovative addition to this model. Art therapy has functioned to support treatment plans, enhance patient experience and reduce the distress associated with receiving treatment for an eating disorder. This presentation outlines the way in which art therapy is practiced in the acute medical environment and how this contributes to quality of care throughout the treatment pathway.

## P17 Who is vulnerable to developing eating disorder symptoms in the context of interpersonal challenges?

### *Katelin Bullman^1^, Elizabeth Rieger^1^, Richard Burns^2^, Kristen Murray^1^

#### ^1^School of Medicine and Psychology, The Australian National University (ANU), Acton, ACT, Australia; ^2^National Centre for Epidemiology and Population Health, ANU, Canberra, ACT, Australia

##### Correspondence: katelin.bullman@anu.edu.au

*Journal of Eating Disorders* 2024, 12(suppl 2): P17

There is a large evidence base supporting the role of interpersonal difficulties as causal factors for eating disorders. However, since not all individuals who experience interpersonal problems develop eating disorder symptoms, it is essential to identify the individual vulnerability factors that may play a moderating role in the relationship between interpersonal problems and eating disorder pathology. The present study sought to address this research gap by providing a comprehensive evaluation of a range of potential moderators of this relationship, namely, shape and/or weight-based self-worth, outcome expectancies regarding eating, shape and weight, and difficulties in emotion regulation. The sample was comprised of women between the ages of 18 to 30 years (N = 1049). Participants completed online self-report questionnaires assessing interpersonal difficulties (Thwarted Belongingness Scale), eating disorder symptoms (Eating Disorder Examination Questionnaire), shape/weight-based self-worth (Body Weight Contingency of Self-Worth Scale), eating outcome expectancies (Eating Expectancy Inventory), shape/weight outcome expectancies (Thinness and Restricting Expectancy Inventory), and emotion dysregulation (Difficulties in Emotion Regulation Scale). The data was analysed using structural equation modelling. Results and clinical implications will be discussed, with a focus on how identifying these moderating factors will help to inform both prevention and treatment approaches for eating disorders.

## P18 Conceptualisation of severe and enduring anorexia nervosa: A qualitative *meta*-synthesis

### *Laura Kiely^1^, Janet Conti^1^, Phillipa Hay^1^

#### ^1^Western Sydney University, NSW, Australia

##### Correspondence: laura.kiely@iinet.net.au

*Journal of Eating Disorders* 2024, 12(suppl 2): P18

Link: Conceptualisation of severe and enduring anorexia nervosa: a qualitative meta-synthesis | BMC Psychiatry | Full Text (biomedcentral.com). https://bmcpsychiatry.biomedcentral.com/articles/10.1186/s12888-023-05098-9.

## P19 Intuitive Exercise Scale (IEXS): analysis of the factorial structure and the psychometric properties of an Italian version

### *Paolo Mancin^1^, Marta Ghisi^1^, Stefania Pittelli^1^, Alexia Dal Pan^1^, Federica Sassano^1^, Martina Rapisarda^1^, Silvia Cerea^1^

#### ^1^Department of General Psychology, University of Padova, Padova, Italy

##### Correspondence: paolo.mancin@phd.unipd.it

*Journal of Eating Disorders* 2024, 12(suppl 2): P19

Intuitive exercise includes the ability to attend to bodily cues, be mindful during movement, and use diverse movement patterns (Reel et al., 2016). The measure utilized to assess it is the Intuitive Exercise Scale (IEXS; Reel et al., 2016), validated for English-speakers. However, an evaluation of the IEXS in other sociocultural contexts, such as Italy, is still lacking. Thus, the present study aimed to examine the factorial structure, gender invariance, internal consistency, and convergent validity of an Italian translation of the IEXS. Moreover, we explored whether intuitive exercise might be related to gender and physical activity. In a sample of 1258 participants (54% women; age: M = 43.59, SD = 12.70; 61% performing physical activities), we highlighted a four-factor structure as adequate, replicating the factorial structure for non-clinical individuals already described for English-speakers. Moreover, gender invariance was supported and internal consistency was adequate. The four subscales demonstrated small-to-moderate correlations with intuitive eating, body appreciation, and body functionality appreciation. In individuals practicing physical activities, small correlations emerged with exercise addiction symptoms, except for emotional exercise that demonstrated a strong positive correlation. Emotional exercise, body trust, and exercise rigidity were associated with practicing physical activities, but not with gender. On the opposite, mindful exercise was associated with gender (i.e., being women), but not with practicing physical activities. The current findings corroborated the four-factor structure of the IEXS for Italian-speakers, as well as its internal consistency, gender invariance, and convergent validity. Finally, practicing physical activities seemed to be associated with reporting higher levels of intuitive exercising.

## P20 Cortical and subcortical networks underlying the processing of negative beliefs related to the self, food, and body image

### *Po-Han Kung^1,2^, Eva Guerrero-Hreins^3,4^, Ben Harrison^2^, Kim Felmingham^1^, Holly Carey^2^, Matthew Greaves^2^, Priya Sumithran^5,6^, Robyn Brown^4^, Trevor Steward^1,2^

#### ^1^Melbourne School of Psychological Sciences, Faculty of Medicine, Dentistry and Health Sciences, University of Melbourne, VIC, Australia; ^2^Melbourne Neuropsychiatry Centre, Department of Psychiatry, University of Melbourne, VIC, Australia; ^3^Department of Biochemistry and Pharmacology, University of Melbourne, VIC, Australia; ^4^Florey Institute of Neuroscience and Mental Health, University of Melbourne, VIC, Australia; ^5^Monash University, VIC, Australia; ^6^Department of Endocrinology, Austin Health, VIC, Australia

##### Correspondence: pkung@student.unimelb.edu.au

*Journal of Eating Disorders* 2024, 12(suppl 2): P20

Negative beliefs about the self, food, and body image contribute to the emotional disturbances that precipitate disordered eating behaviours. Psychotherapy for eating disorders often seek to restructure these maladaptive cognitions. Despite its clinical relevance, the neurobiological substrates underpinning cognitive restructuring remain unclear. Combining ultra-high field 7-Tesla magnetic resonance imaging and a novel cognitive restructuring paradigm, we explored cortical and subcortical involvement during the repeating or challenging of negative self- and disordered eating-related beliefs in 48 healthy adults (47.9% female, mean age = 25.8). We confirmed that self-reported negative belief endorsement positively correlated with measures of psychological distress (r = 0.53, p < 0.001), repetitive negative thinking (r = 0.65, p < 0.001), and disordered eating (r = 0.65, p < 0.001). Challenging relative to repeating negative beliefs elicited increased activation in frontostriatal cognitive control regions, including the pre-supplementary motor area and the caudate. Whereas repeating negative beliefs was associated with heightened activity in default mode network regions linked to self-related processes (e.g., dorsal posterior cingulate cortex, ventromedial prefrontal cortex), and the habenula – a pair of midbrain nuclei implicated in negative event processing and the regulation of food intake. The present study characterises key cortical and subcortical regions involved in the processing of negative self- and disordered eating-related beliefs. Notably, we identified the first evidence in humans to demonstrate habenular involvement in processing negative cognitions, laying the foundation for future work to examine potential habenula alterations in people with disordered eating.

## P21 Identifying Key Elements of Eating Disorder Services

### *Rachel Knight^1^, Genevieve Pepin^1^

#### ^1^Deakin University, Burwood, VIC, Australia

##### Correspondence: rachel.knight@deakin.edu.au

*Journal of Eating Disorders* 2024, 12(suppl 2): P21

In Australia, eating disorder (ED) service models vary greatly. Services can be publicly or privately funded or only available to people of specific ages or diagnosis. Because of these differences, navigating services for eating disorders is often confusing and complex, delaying much needed care. This research aimed to identify the essential components of a comprehensive service model and explore the acceptability of a proposed integrated approach to service delivery for eating disorders. This study was part of a larger research project. Results from a previous qualitative study combined with review of relevant literature informed the development of the proposed integrated service model. In this study, agreement on the essential components and consensus on the integrated model was achieved using the Delphi technique. Three rounds of surveys were completed by three groups of participants: public mental health clinicians, carers, and previous service users. Consensus was reached for 43 of the original 52 statements. A score of 100% consensus was reached for seven statements, including ‘a need for more clinicians with ED expertise’, ‘reduced wait lists’ and ‘the inclusion of both medical and mental health care’. This study has sought agreement on an integrated model of service delivery for eating disorders. It has also identified 43 essential components of service models for eating disorders. Inclusion of these components in current and future ED services may enhance effectiveness and efficiency. The results of this study suggest that in future, ED services need to be more responsive, flexible and person centred.

## P22 A systematic review of exercise interventions for patients with Eating Disorder

### *Ryan Bell^1^, Sau Man Yiu^1^, Wendy Brown^2,3^, Sabine Woerwag-mehta Woerwag-Mehta^1,2^

#### ^1^Gold Coast Health Service, Southport, QLD, Australia; ^2^Queensland 2 Bond University, Robina, QLD, Australia; ^3^University of Queensland, St Lucia, QLD, Australia

##### Correspondence: ryan.bell2@health.qld.gov.au

*Journal of Eating Disorders* 2024, 12(suppl 2): P22

Aim: To understand the safety, benefits and limitations of exercise interventions for patients with eating disorders.

Objective: To systematically review the evidence from randomised controlled trials examining the efficacy and safety of exercise interventions for people with eating disorders.

Methods: Electronic databases (including CINAHL, Cochrane CENTRAL, Embase, Medline, PsychInfo, and WebofScience) were searched from 2013 to 2022. Articles were eligible if they utilised a randomised control trial design to compare various types of exercise therapy with care as usual or waitlisted control and included patients with Anorexia Nervosa, Bulimia Nervosa, Binge Eating Disorder, or Eating Disorder Not Otherwise Specified. Methodological quality was assessed using the Tool for the assEssment of Study qualiTy and reporting in EXercise (TESTEX).

Results: Twelve RCTs involving 653 (332 intervention vs 321 control) participants met selection criteria with eleven studies having strong methodological quality (TESTX score > 10). Studies tested aerobic, strength based or a combination of both types of exercise intervention and reported improvement in at least one major, physiological, or wellbeing domain while no adverse effects were reported.

Conclusions: Aerobic and strength based exercise have beneficial outcomes on various aspects of all major eating disorder pathologies. This systematic review additionally provided evidence that the application and use of physical therapy interventions does not appear to cause or worsen injury or pathology when used with patients with eating disorder pathology.

## P23 Individualised Harm Reduction and Somatic therapies—working with complex presentations and treatment trauma

### *Sandi James^1,2^

#### ^1^Sandi James Psychology, Sunshine Coast, QLD, Australia; ^2^Western Sydney University, Penrith, NSW, Australia

##### Correspondence: sandijamespsych@gmail.com

*Journal of Eating Disorders* 2024, 12(suppl 2): P23

Disordered eating, eating disorders, and body image concerns continue to escalate alongside increases in substance use disorders and trauma related presentations. The isolation and disconnection precipitated by the pandemic has seen demand for specialised and compassionate care explode. The complexity of treating co-occurring conditions is not a new phenomena. Abstinence based approaches focused on treating one presenting condition can cause harm for those experiencing symptoms that cross more than one diagnostic label. Harm reduction is not a new concept. Implementing harm reduction and trauma informed approaches to treating eating disorders does seem to be relatively new and is gaining traction in the field. This presentation will explore approaches currently dominating eating disorder treatment at all levels of care and offer 'food for thought' around what can be done differently to provide more effective care with those for whom standard models of care are failing. Collaborating with individuals and families to formulate an approach that includes the needs and goals of each person, while also supporting the connection and growth offered through group based programs, is challenging. Harm reduction approaches that support recovery from multiple mental and physical conditions needs further exploration. Ensuring everyone seeking help for an eating disorder gets effective and accessible assessment and treatment for co-occurring disorders is emerging as an essential area of focus for researchers and clinicians alike. As a clinician with lived experience, discussion will give insight from multiple perspectives and include preliminary findings from a PhD study exploring treatment trauma in this discrete population.

## P24 Scoping Review: Global Prevalence And Educational Strategies To Address Eating Disorders And Predictors Of Eating Disorders In Nutrition And Dietetic University Students

### *Sarah Verina Budhiwianto^1^, Christie Bennett^1^, Claire Bristow^2^, Janeane Dart^1^

#### ^1^Department of Nutrition, Dietetics and Food, School of Clinical Sciences, Monash University, Melbourne, VIC, Australia; ^2^Medical Education and Research Quality Unit (MERQ). School of Public Health and Preventive Medicine, Monash University, Melbourne, VIC, Australia

##### Correspondence: sarah.budhiwianto@monash.edu

*Journal of Eating Disorders* 2024, 12(suppl 2): P24

LINK: Global Prevalence of Eating Disorders in Nutrition and Dietetic University Students: A Systematic Scoping Review—PMC (nih.gov). https://www.ncbi.nlm.nih.gov/pmc/articles/PMC10221384/.

## P25 A *Meta*-Analysis of Disordered Eating and its Association with Self-Criticism and Self-Compassion

### *Sarah Paranjothy^1^, Tracey Wade.^1^

#### ^1^Flinders University Institute for Mental Health and Wellbeing, Bedford Park, SA, Australia

##### Correspondence: para0118@flinders.edu.au

*Journal of Eating Disorders* 2024, 12(suppl 2): P25

LINK: A meta‐analysis of disordered eating and its association with self‐criticism and self‐compassion—Paranjothy—2024—International Journal of Eating Disorders—Wiley Online Library. https://onlinelibrary.wiley.com/doi/10.1002/eat.24166.

## P26 Exploring the Relationship Between Self-Compassion and Positive Body Image Construct

### *Si Woo Chae^1^

#### ^1^University of Hawaii, Manoa, Honolulu, Hawaii

##### Correspondence: swchae@hawaii.edu

*Journal of Eating Disorders* 2024, 12(suppl 2): P26

Positive body image has emerged as a construct distinct from negative body image. Studies have indicated that reducing negative body image distress does not necessarily lead to boosting acceptance of or appreciation and respect for one’s body. Self-compassion has been introduced as a potentially beneficial approach in promoting a more accepting and kind attitude towards one’s flaws, including physical imperfections. The present study aimed to explore the relationship between self-compassion and positive body image, specifically body functionality appreciation. A total of 270 undergraduate women completed the Self-Compassion Scale (SCS) and Body Functionality Appreciation Scale (FAS). Multiple linear regression analyses were conducted to examine the relationship between self-compassion and body functionality appreciation. The results indicated a significant main effect of body functionality appreciation on self-compassion (β = 0.35, p < 0.001). Particularly, the FAS scores significantly predicted one’s tendency to show kindness (β = 0.18, p = 0.006), recognize common humanity (β = 0.25, p < 0.001), and embrace mindfulness (β = 0.23, p = 0.001). Interestingly, the FAS scores also predicted scores on self-judgment (β = 0.60, p < 0.001) and over-identification of imperfections (β = 0.48, p < 0.001). The findings suggest positive and negative poles of self-compassion are not mutually exclusive, consistent with previous studies that indicated an individual may embrace both perspectives. It is also possible that the distinct construct of positive body image is primarily associated with enhancing one’s positive attitude towards one’s body image rather than reducing body image distress.

## P27 Positive vs. Negative Body Image Constructs: Differing Patterns of Relationship on Appearance Evaluation and Appearance Orientation

### *Si Woo Chae

#### ^1^University of Hawaii, Manoa, Honolulu, Hawaii

##### Correspondence: swchae@hawaii.edu

*Journal of Eating Disorders* 2024, **12**(**suppl 2**): P27

Since the positive psychology movement, which asserts that reducing illness does not necessarily enhance wellness, emphasis on positive body image has been growing. However, while several studies have examined the relationship between body image distress and how an individual thinks about and evaluates her own body, only a few attempts have been made to explore such relationships in the context of positive body image. The present study aimed to explore appearance evaluation and orientation, and their relationship with other positive and negative body image constructs. A total of 270 undergraduate women completed the Multidimensional Body-Self Relations Questionnaire (MBSRQ), Functionality Appreciation Scale (FAS), Body Appreciation Scale (BAS-2), Body Shape Questionnaire (BSQ-8), and Objectified Body Consciousness Scale (OBCS). Multiple linear regression analyses were conducted to examine the relationship of appearance evaluation and orientation to positive and negative body image constructs. The results indicated that body appreciation (β = 0.64, p < 0.001), body surveillance (β = 0.09, p = 0.084), body shame (β = -0.30, p < 0.001), and body dissatisfaction (β = -0.37, p < 0.001) significantly predicted appearance evaluation. Body dissatisfaction (β = -0.37, p < 0.001) and body shame (β = 0.18, p = 0.010) significantly predicted appearance orientation. Findings suggest that appearance evaluation is associated with both positive and negative aspects of body image, while appearance orientation is only associated with negative aspects of body image. Both domains should be considered in order to gain a comprehensive understanding of feelings and thoughts about one’s own body.

## P28 A systematic review of educational training provided to health professionals assessing and managing patients with eating disorders

### *Stephanie Sardinha^1^, Grace Branjerdporn^2^, Sabine Woerwag-Mehta^1,2^

#### ^1^Bond University, Robina, QLD, Australia; ^2^Gold Coast Health, Gold Coast, QLD, Australia

##### Correspondence: stephaniesardinha555@gmail.com

*Journal of Eating Disorders* 2024, 12(suppl 2): P28

Background: Eating disorders (ED) are potentially life-threatening mental illnesses; many mental health clinicians have limited knowledge and understanding. Continuing education is pivotal in ensuring that health practitioners keep up to date with evidence-based practices. 1

The aim of this systematic review is to (1) synthesise and summarise the level of education provided about ED to healthcare workers and (2) analyse the effectiveness of these education modalities.

Methodology: Databases (e.g. CINAHL, PubMed, PsychInfo, Scopus, Web of Science) were searched systematically using a search strategy comprising of concepts related to eating disorders, education and health professionals. Covidence was used for title and abstract screening, full text review and data extraction. QuADS assessment tool was used for quality appraisal. 2

Results: Low levels of confidence was reported consistently across all studies, often due to lack of clinical guidelines, formal training and clinical exposure. Studies also reported low levels of knowledge across health disciplines, with the exception of those who specialise in eating disorders.

Three studies evaluated the improvement in knowledge and skills after completion of an educational modality, with interprofessional education and online training found to be helpful.

Conclusions: Results indicate that there are low levels of confidence and knowledge that warrant increased training on this topic. Scant research has explored the effectiveness of ED education modalities. Further research is required to examine the potential effectiveness of novel educational modalities, such as simulation and virtual reality, to support greater knowledge and improved practical clinical skills to manage ED.

